# Optimization of process parameters for enhancing the tensile strength of diffusion-bonded Al–Al and Al–Fe–Al laminated structures

**DOI:** 10.1371/journal.pone.0354878

**Published:** 2026-08-11

**Authors:** An-Le Van, Nguyen Truong Sinh, Bui Chan Thanh, Tran Minh The Uyen, Pham Son Minh

**Affiliations:** 1 Faculty of Engineering and Technology, Nguyen Tat Thanh University, Ho Chi Minh City, Viet Nam; 2 Faculty of Mechanical Engineering, Ho Chi Minh City University of Technology and Engineering, Ho Chi Minh City, Vietnam; 3 Faculty of Mechanical Engineering, Ho Chi Minh City University of Technology and Engineering, Ho Chi Minh City, Vietnam; Galgotias University, INDIA

## Abstract

Diffusion bonding has been widely investigated for dissimilar metal systems such as aluminum–steel and aluminum–titanium joints. However, systematic studies on the hot-press bonding of similar aluminum sheet joints under controlled processing conditions remain relatively limited. Furthermore, although aluminum–steel bonding has been extensively studied, research on Al–Fe–Al laminated structures, in which steel serves as a load-bearing core and aluminum acts as a protective outer layer, is still scarce. In particular, limited attention has been given to the simultaneous optimization of bonding conditions for both Al–Al and Al–Fe–Al structures within the same experimental framework. In addition, the bonding strength of diffusion-bonded laminated structures is strongly influenced by multiple processing parameters, yet the combined effects of these parameters have not been systematically optimized for dual-material laminated systems. Therefore, this study aims to investigate the hot-press bonding process of Al–Al and Al–Fe–Al stacked structures and to optimize the processing parameters to achieve improved tensile performance. In this work, the effects of pressing temperature, pressing force, holding time, cooling rate, and heating power on the bonding quality were investigated using a Box–Behnken Design. The tensile strength of Al–Al and Al–Fe–Al structures was selected as the primary response for evaluating the bonding performance. Response Surface Methodology was used to develop predictive models describing the relationship between the processing parameters and the tensile strength responses. Subsequently, multi-objective optimization using the NSGA-II algorithm was applied to determine the optimal processing conditions for maximizing the tensile strength of both bonded material systems simultaneously. The results demonstrate that the developed models can effectively describe the process–property relationships and identify a set of optimal hot-pressing parameters for improving the tensile strength of the bonded structures. Scanning electron microscopy was used as a supporting method to qualitatively examine the interfacial morphology and fracture features after bonding and tensile testing. The findings provide a process-oriented optimization framework for improving the mechanical performance of aluminum-based laminated structures and offer practical guidance for selecting hot-press bonding conditions for Al–Al and Al–Fe–Al systems.

## Introduction

Aluminum and its alloys have been widely used in various engineering fields, including aerospace, automotive, and thermal management systems, due to their low density, good corrosion resistance, high thermal conductivity, and favorable mechanical properties [1–5]. Recent studies have continued to emphasize the importance of improving the mechanical performance of aluminum-based materials for lightweight structural applications. For example, combined electromagnetic agitation and grain refinement have been shown to enhance the strength, hardness, elongation, and wear resistance of Al-Si7Mg alloy, highlighting the continuing demand for process-based strengthening strategies in aluminum systems [6]. In addition, recent reviews on aluminum-based metal matrix composites have shown that advanced processing and optimization approaches are increasingly important for tailoring the microstructure and mechanical properties of aluminum-based materials for high-performance applications [7]. Laminated metal structures have attracted significant attention for many years, and they continue to be extensively studied due to their ability to combine different materials to achieve a balance of mechanical strength, lightweight characteristics, and functional performance [8–12]. Among these structures, aluminum–steel laminated composites have attracted significant interest because they integrate the high strength and stiffness of steel with the lightweight and corrosion-resistant properties of aluminum [13–17]. Such hybrid structures are promising for applications requiring both structural reliability and weight reduction. Therefore, understanding the fabrication and bonding behavior of aluminum-based laminated materials is important for expanding their applications in advanced engineering systems.

Joining aluminum components, particularly thin sheets, presents several technical challenges when conventional fusion welding methods are employed [18,19]. Aluminum surfaces are naturally covered by a stable oxide layer (Al_2_O_3_), which has a much higher melting temperature than the base metal and can hinder proper fusion during welding [20,21]. In addition, aluminum exhibits high thermal conductivity and a large thermal expansion coefficient, which often lead to rapid heat dissipation, weld pool instability, distortion, and the formation of defects such as porosity and incomplete fusion [22]. These issues become more pronounced when welding thin aluminum sheets, where burn-through and dimensional inaccuracies may occur. To overcome these limitations, diffusion bonding has been considered a promising alternative joining technique [23–25]. As a solid-state bonding process, diffusion bonding joins materials at elevated temperature and pressure without melting the base metal, thereby minimizing thermal distortion and reducing welding defects. This method can produce joints with uniform bonding across large contact areas and has been increasingly explored for the fabrication of high-quality layered and laminated metallic structures [26–28].

Solid-state joining technologies have recently received renewed attention because they can reduce melting-related defects and provide better control of bonding quality in similar and dissimilar metallic systems. Roll bonding, for instance, has been widely used for joining similar and dissimilar metal sheets through severe plastic deformation and pressure, and its bonding quality is strongly related to surface preparation, pressure, temperature, and deformation history [29]. Friction-based solid-state processes have also been investigated for Al–Fe joining because they can reduce heat input and limit the formation of detrimental interfacial products compared with fusion-based methods [30]. More recently, large-area solid-state additive manufacturing and additive friction stir deposition have demonstrated the possibility of achieving high-strength Al–Fe bonding by carefully controlling the interfacial structure [31, 32]. These studies confirm the modern relevance of solid-state joining for Al–Fe and aluminum-based laminated structures. Nevertheless, many of these techniques require specialized equipment, tool motion, severe plastic deformation, or complex deposition paths, whereas hot-press diffusion bonding remains attractive because it allows controlled temperature, pressure, and holding time to be applied over a relatively large contact area.

Previous studies have investigated the diffusion bonding behavior of aluminum and its alloys in order to understand the mechanisms governing interfacial bonding and joint performance [33,34]. Researchers have reported that successful Al–Al diffusion bonding can be achieved when appropriate combinations of temperature, pressure, and holding time are applied to promote atomic diffusion across the interface [35,36]. Several studies have focused on the microstructural evolution at the bonding interface, including grain growth, void elimination, and the disruption of the surface oxide layer during the bonding process [37,38]. It has been shown that the bonding temperature plays a critical role in enhancing atomic mobility, while sufficient pressure helps increase the real contact area between the mating surfaces [39,40]. Although these studies provide valuable insights into the bonding mechanisms of aluminum materials, many of them primarily emphasize microstructural characterization [35,41], while relatively fewer investigations have systematically evaluated the mechanical performance of diffusion-bonded Al–Al joints, particularly in terms of tensile strength and the influence of multiple processing parameters.

Extensive research has also been conducted on the bonding between aluminum and steel due to the potential advantages of combining the lightweight characteristics of aluminum with the high strength and stiffness of steel. Various joining techniques, including welding, brazing, and diffusion bonding, have been explored to fabricate Al–steel hybrid structures [42–44]. Among these methods, diffusion bonding has attracted considerable attention because it can produce joints with minimal thermal distortion and good interfacial integrity. Previous studies have reported that during the bonding process, intermetallic compounds such as Fe_2_Al₅ and FeAl_3_ may form at the aluminum–steel interface due to interdiffusion between the two metals. The thickness and morphology of these intermetallic layers significantly influence the mechanical properties of the bonded joint, as excessive growth of brittle intermetallic phases can reduce joint strength [45,46]. The mechanical behavior and bonding characteristics of Fe–Al intermetallic phases have also been studied from both experimental and theoretical viewpoints, showing that different Fe–Al phases can exhibit different stability, hardness, and brittleness [47,48]. In addition, recent Al/Fe composite studies have shown that the effect of intermetallic compounds on bonding strength is not determined only by their presence, but also by their thickness, continuity, morphology, and associated crack formation [49]. Therefore, controlling the interfacial condition remains a critical issue in Al–Fe bonding.

Several strategies have been proposed to improve Al–Fe bonding by modifying the interfacial reaction or stress state. For example, gallium-assisted diffusion bonding has been used to promote interfacial bonding between aluminum and steel, with SEM-EDX and XRD used to examine interfacial reaction products [50]. Other studies have introduced Ni-based interlayers to modify the reaction layer and reduce interfacial stress concentration, thereby improving bonding strength in Al/Fe bimetallic systems [51]. Recent friction-based studies have also attempted to suppress or control brittle intermetallic formation at the Al–Fe interface [30–32]. These studies demonstrate that interfacial phenomena are important in Al–Fe bonding. However, most of them focus on direct Al–Fe interfaces, interlayer-assisted bonding, or friction-based joining routes. Relatively limited attention has been given to hot-press diffusion-bonded Al–Fe–Al laminated structures in which steel serves as a core layer and aluminum acts as the outer layers.

In parallel with advances in solid-state joining, process optimization methods have become increasingly important for improving manufacturing quality and reducing experimental cost. Response Surface Methodology (RSM), Box–Behnken Design (BBD), Taguchi methods, Grey Relational Analysis (GRA), TOPSIS, Genetic Algorithms (GA), Particle Swarm Optimization (PSO), and Artificial Neural Networks (ANN) have been widely applied to model and optimize complex process–property relationships. For example, RSM combined with GRA and TOPSIS has been applied to optimize friction stir welding parameters for dissimilar aluminum alloys, showing that hybrid decision-making methods can effectively balance tensile strength, elongation, and hardness [52]. ANN-based optimization has also been applied to welding processes to capture nonlinear relationships between input parameters and weld quality responses [53]. Similarly, BBD–RSM has been used to model and optimize thermal performance in heat pipe systems [54], while GA, PSO, and ANN-based approaches have been used to improve prediction and optimization accuracy in machining applications [55,56]. These recent works indicate that optimization tools are now widely used in manufacturing research. Therefore, the novelty of a new study should not rely only on the use of an optimization algorithm, but rather on the material system, process conditions, response selection, and engineering problem being addressed.

Despite the progress reported in previous studies, several issues remain insufficiently addressed. While diffusion bonding has been extensively studied for dissimilar metal systems, particularly aluminum–steel joints, comparatively less attention has been given to the bonding behavior of similar aluminum materials, especially in terms of their mechanical performance under different processing conditions. In addition, most existing investigations on aluminum–steel systems focus on direct Al–steel interfaces, whereas studies involving laminated configurations such as Al–Fe–Al structures are still limited. Furthermore, the quality and mechanical strength of structures are strongly influenced by multiple processing parameters, including temperature, pressure, and holding time, yet their combined effects have not been systematically evaluated. In particular, few studies have simultaneously considered Al–Al and Al–Fe–Al bonded structures within the same hot-press diffusion bonding framework and optimized their tensile performance together. As a result, a comprehensive investigation that compares Al–Al bonding and Al–Fe–Al laminated structures under controlled diffusion bonding conditions, while systematically analyzing the influence of processing parameters on tensile performance, remains necessary.

**Fig 1:**
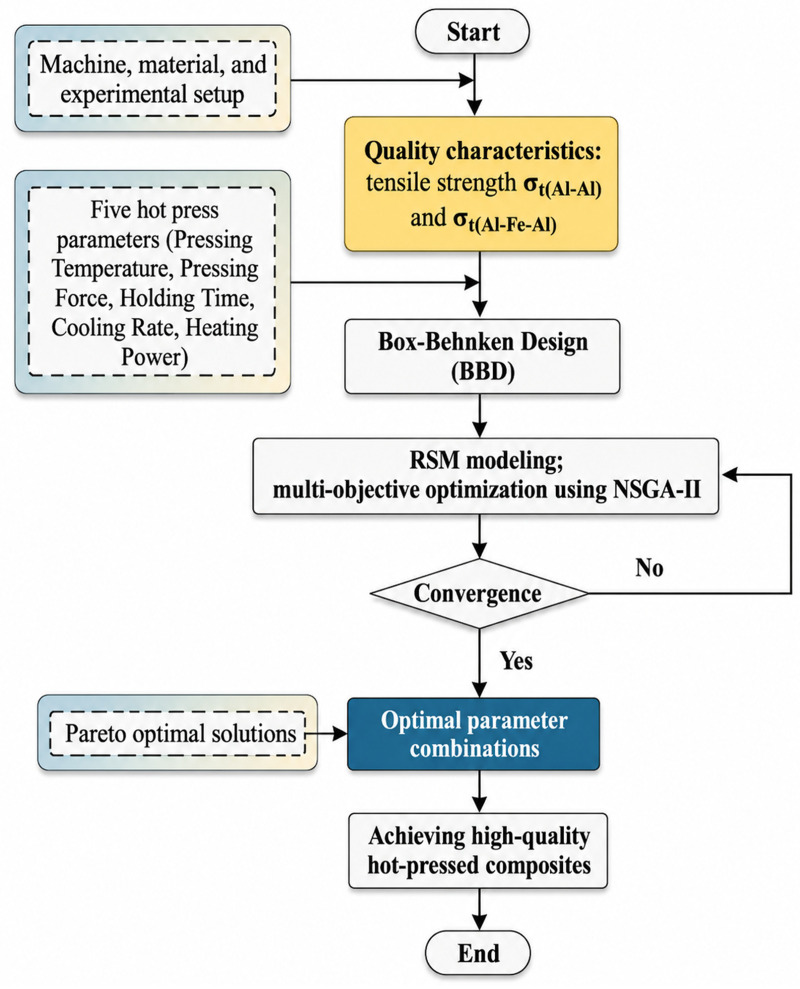
Workflow of the Box–Behnken design–RSM NSGA-II optimization approach for improving the tensile performance of hot-pressed Al–Al and Al–Fe–Al composites.

Therefore, this study aims to investigate the diffusion bonding behavior of Al–Al and Al–Fe–Al stacked structures and to determine the optimal processing conditions that improve their tensile performance. The overall research methodology follows the workflow illustrated in Fig 1, which presents the integration of experimental design, modeling, and optimization. In the hot-press diffusion bonding process, key processing parameters including pressing temperature, pressing force, holding time, cooling rate, and heating power were selected as input variables. The tensile strengths of the Al–Al and Al–Fe–Al laminated structures were used as the primary responses to evaluate the bonding quality. A Box–Behnken Design (BBD) was first employed to design the experimental matrix and systematically investigate the influence of the processing parameters. Based on the experimental results, Response Surface Methodology (RSM) was applied to establish predictive models describing the relationships between the processing parameters and the tensile strength responses. Subsequently, a multi-objective optimization approach using the NSGA-II algorithm was implemented to identify the optimal combinations of processing parameters that simultaneously enhance the tensile performance of both bonding material systems. Furthermore, scanning electron microscopy (SEM) was conducted to qualitatively examine the interfacial morphology and fracture characteristics after bonding and tensile testing. In this study, SEM analysis was used as supporting evidence for fracture and interfacial morphology, while detailed phase identification and diffusion-layer thickness measurement are recognized as future research directions. The main contribution of this work is the application of an integrated experimental–statistical–optimization framework to a dual laminated bonding problem involving both Al–Al and Al–Fe–Al structures under the same hot-press diffusion bonding conditions.

## Experimental section

### Materials

In this study, aluminum alloy A6061 and carbon steel SS400 were used as the base materials for the diffusion bonding experiments. Aluminum alloy A6061 was selected as the outer material because of its low density, good corrosion resistance, and favorable mechanical properties, which make it widely used in structural and engineering applications. Steel SS400 was employed as the core material in the Al–Fe–Al laminated structure because of its relatively high strength and stiffness, which can enhance the load-bearing capability of the composite structure.

**Fig 2:**
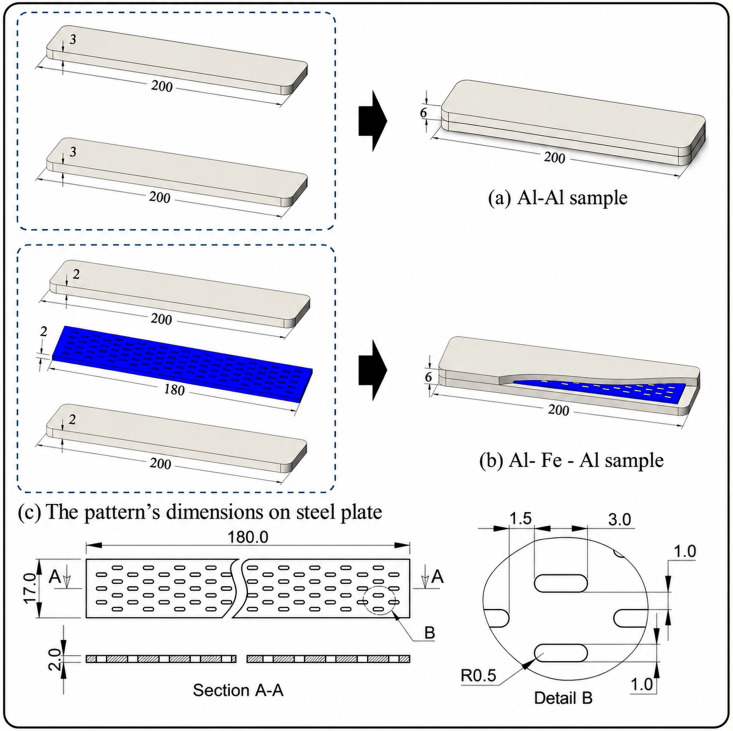
Preparation process of (a) Al-Al, (b) Al-Fe-Al workpiece sample and (c) Pattern’s dimension of steel plate.

For the Al–Al bonding case, two A6061 aluminum specimens were stacked together to form a direct aluminum–aluminum bonding interface. The preparation procedure and the geometry of the Al–Al specimens are illustrated in Fig 2(a), where the specimen size and bonding configuration are presented.

For the Al–Fe–Al laminated configuration, a steel SS400 plate was placed between two A6061 aluminum plates, forming a sandwich structure in which the steel acted as the core layer and the aluminum plates served as the outer cladding layers. The steel layer was designed with a slightly smaller size than the aluminum plates in order to allow the aluminum layers to cover the steel core during the hot-press bonding process. In addition, a hole-pattern structure was machined on the steel layer, as shown in Fig 2(b). The pattern on steel plate was illustrated in Fig 2(c). This patterned structure was introduced to increase the effective contact area between the aluminum and steel layers and to promote mechanical interlocking during hot pressing. Under elevated temperature and compressive force, the softened aluminum can locally deform into the patterned holes, thereby improving the physical constraint between the aluminum layers and the steel core.

Prior to the bonding process, the contact surfaces of all specimens were mechanically polished and cleaned with acetone to remove surface contaminants and reduce the influence of surface impurities on the bonding interface. After cleaning, the prepared specimens were stacked according to the designed Al–Al and Al–Fe–Al configurations and then subjected to the hot-press diffusion bonding process under controlled processing parameters, including pressing temperature, pressing force, holding time, cooling rate, and heating power.

### Experiment design

In this study, the experimental plan was developed using the Box–Behnken design (BBD), which is one of the most widely used experimental designs in Response Surface Methodology (RSM) for modeling and optimization of engineering processes [57]. BBD is particularly suitable for investigating the relationships between multiple process variables and response values when the objective is to establish a quadratic regression model with a reduced number of experiments compared with a full factorial design. In addition, this design is efficient because it avoids experimental combinations at the extreme corners of the design space, thereby reducing the risk of conducting impractical or unstable test conditions while still providing sufficient information to evaluate the main effects, interaction effects, and quadratic effects of the selected factors. Owing to these advantages, BBD has been broadly applied in manufacturing, materials processing, and process optimization studies.

Five processing parameters were selected as the input factors in the present work, namely pressing temperature (A), pressing force (B), holding time (C), cooling rate (D), and heating power (E). Each factor was investigated at three levels, designated as low (−1), center (0), and high (+1). The coded factors and their corresponding actual values are presented in Table 1. Specifically, the pressing temperature was varied from 450 to 500 °C, the pressing force from 20 to 50 kN, the holding time from 5 to 30 min, the cooling rate from 13 to 17 °C/min, and the heating power from 40 to 70%.

**Table 1:** Processing parameters and their levels used in the Box–Behnken design.

Factor	Parameter	Unit	Low (−1)	Center (0)	High (+1)
**A**	Pressing temperature	°C	450	475	500
**B**	Pressing force	kN	20	35	50
**C**	Holding time	min	5	17.5	30
**D**	Cooling rate	°C/min	13	15	17
**E**	Heating power	%	40	55	70

The selected ranges were determined based on the operating capability of the hot-press system and the need to maintain stable bonding conditions during the experiments. In particular, the cooling rate was varied within a relatively narrow range of 13–17 °C/min because the cooling stage in the hot-press system could be controlled reliably within this window. This range was also selected to avoid excessive thermal gradients during post-bonding cooling, which may cause additional residual stress or distortion in the bonded specimens. Although the cooling-rate window was narrower than those of the other factors, it was included in the design because cooling behavior can still influence the final tensile performance of diffusion-bonded structures, especially in laminated material systems.

For a BBD with five factors, the number of experimental runs is calculated as follows:


$$\begin{array}{c} {N=2k(k-1)+C}_{0} \end{array}$$

where k is the number of input factors and C₀ is the number of replicated center points. In the present study, k = 5; therefore, the number of non-center design points was 2 × 5 × (5 − 1) = 40. Six center-point experiments were added to estimate pure experimental error and evaluate the repeatability and stability of the bonding process. As a result, the total number of experimental runs was 40 + 6 = 46. The replicated center points corresponded to the middle levels of all five parameters: pressing temperature of 475 °C, pressing force of 35 kN, holding time of 17.5 min, cooling rate of 15 °C/min, and heating power of 55%.

After conducting the experiments, the obtained tensile strength results were analyzed using Response Surface Methodology to develop predictive models correlating the processing parameters with the tensile strength responses of the Al–Al and Al–Fe–Al specimens. Analysis of variance (ANOVA) was then performed to evaluate the statistical significance of the model terms, identify the most influential parameters, and assess the adequacy of the developed regression models. The replicated center-point data were used to estimate pure error, which is necessary for evaluating the lack-of-fit of the regression models. Based on the developed regression equations, multi-objective optimization using the NSGA-II algorithm was subsequently employed to determine the optimal combination of process parameters for simultaneously improving the tensile strength of both bonded structures.

### Specimen processing

**Fig 3:**
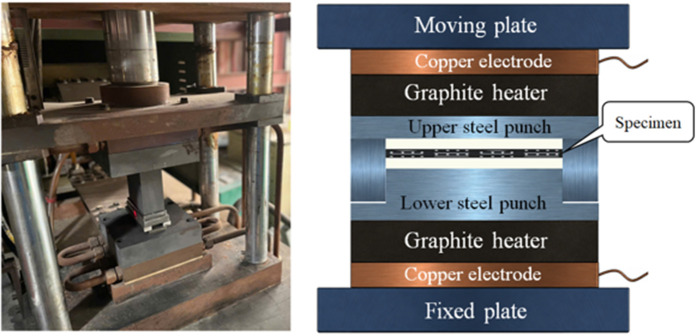
The configuration of the hot press machine.

The diffusion bonding experiments were carried out using a hot-press sintering machine (Model SJJ-HXP), and the configuration of the machine as well as the bonding process are illustrated in Fig 3. The heating system of the hot press consists of two graphite heaters, which are electrically heated by a transformer through copper electrodes. These graphite heaters are positioned between two structural plates, namely a fixed plate at the bottom and a moving plate at the top, allowing compressive force to be applied during the bonding process.

During operation, the moving plate applies a pressing force to the specimen through the upper steel punch, while the lower steel punch is supported by the fixed plate. The stacked specimens were placed between the upper and lower steel punches, ensuring uniform pressure transfer during the hot-press process.

The hot-press sintering machine used in this study operates with a power supply of 380 V at 50/60 Hz and provides a rated working pressure of up to 125 kN. The heating system has a power range of 30–80 kW, with a temperature control range of 400–1200 °C, which enables precise control of the bonding temperature during the process. The machine has an opening height of 250 mm and a workbench area of 200 mm × 200 mm, which is sufficient to accommodate the specimen assembly and die system used in this study. The overall dimensions of the equipment are 820 mm × 1450 mm × 2100 mm, with a total weight of approximately 1500 kg. The specifications are shown in Table 2.

**Table 2:** Specifications of the hot-press sintering machine (Model SJJ-HXP).

Parameter	Specification
**Model**	SJJ-HXP
**Power supply**	380 V, 50/60 Hz
**Rated working pressure**	0–125 kN
**Heating power**	30–80 kW
**Temperature control range**	400–1200 °C
**Opening height**	250 mm
**Workbench area**	200 mm × 200 mm
**Machine dimensions**	820 mm × 1450 mm × 2100 mm
**Machine weight**	1500 kg

**Fig 4:**
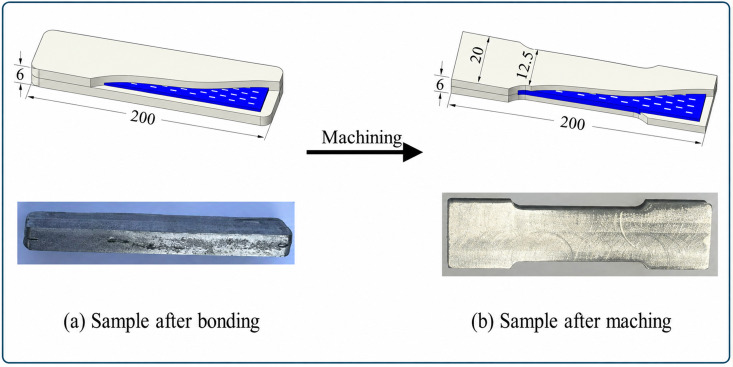
The testing sample with (a) Sample after bonding and (b) Tensile specimens after machining.

For the experiments, the prepared Al–Al and Al–Fe–Al stacked specimens were positioned at the center of the steel die between the punches. Once the assembly was properly aligned, electrical power from the transformer was supplied to the graphite heaters to generate heat, while the required compressive force was simultaneously applied through the moving plate. The bonding process was conducted under controlled processing conditions including pressing temperature, pressing force, holding time, cooling rate, and heating power, according to the experimental design. After the bonding stage, the specimens were allowed to cool under controlled conditions before being removed from the die. The specimens after bonding are illustrated in Fig 4(a). The specimens were then CNC-machined in preparation for the tensile test (Fig 4(b)).

### Tensile testing

After completing the hot-press diffusion bonding experiments according to the Box–Behnken experimental design, tensile tests were conducted to evaluate the mechanical performance and bonding quality of the prepared specimens. Tensile tests were performed for both Al–Al bonded samples and Al–Fe–Al laminated structures under the different combinations of processing parameters defined in the experimental matrix.

**Fig 5:**
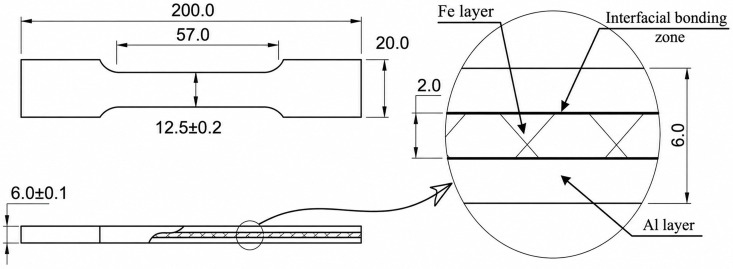
Detailed dimensions of the tensile test specimen.

All tensile tests were carried out in accordance with the ASTM E8/E8M standard test method for tension testing of metallic materials [58]. Based on this standard, the dimensions correspond to the 12.5 mm wide sheet-type specimen geometry in ASTM E8/E8M were used. The specimens were machined using a CNC milling process to ensure dimensional accuracy and consistency. The final specimen dimensions were: overall length (L) = 200 mm, reduced section length (A) = 57 mm, width (W) = 12.5 mm, grip section width (C) = 20 mm, and thickness (T) = 6 mm. Fig 5 shows the specific dimensions of the specimen.

During testing, tensile load was applied along the longitudinal axis of the specimen to ensure uniaxial stress conditions, and careful alignment was maintained to avoid bending effects. The tests were conducted under controlled loading conditions until fracture occurred, and the maximum tensile strength was recorded as the primary response for evaluating the bonding performance of both Al–Al and Al–Fe–Al samples.

According to the ASTM standard, fracture should occur within the gauge section to ensure the validity of the test results. If failure occurs near the grip region, the measured properties may not accurately represent the material behavior and should be disregarded. After tensile testing, selected fractured specimens were further examined using scanning electron microscopy to qualitatively observe fracture features and interfacial morphology.

## Result and discussion

The measured tensile strength values obtained from these experiments are summarized in Table 3, which includes the results from 46 experimental cases corresponding to various processing conditions of pressing temperature, pressing force, holding time, cooling rate, and heating power.

**Table 3:** Experimental design and tensile test results.

No.	A (°C)	B (kN)	C (min)	D (°C/min)	E (%)	σ_t_ (Al–Al) (MPa)	σ_t_ (Al–Fe–Al) (MPa)
**1**	450	35	17.5	15	40	203.6	286.5
**2**	475	35	17.5	13	70	207.6	296.7
**3**	475	35	17.5	15	55	200.3	292.1
**4**	475	35	17.5	15	55	203.7	294.7
**5**	475	20	17.5	15	40	202.8	294.9
**6**	450	35	5	15	55	202.5	289.7
**7**	450	35	30	15	55	200.4	290.4
**8**	475	20	5	15	55	200.8	294.8
**9**	500	35	17.5	13	55	206.3	297.1
**10**	475	35	17.5	15	55	206.8	292.5
**11**	500	20	17.5	15	55	202.1	295.2
**12**	500	35	5	15	55	204.1	296.8
**13**	475	50	17.5	15	70	204.3	296.8
**14**	475	35	5	13	55	201.5	292.7
**15**	500	35	17.5	15	40	208.7	298.0
**16**	475	50	17.5	17	55	206.0	297.6
**17**	475	50	17.5	13	55	204.7	296.4
**18**	475	20	17.5	17	55	196.8	292.7
**19**	475	35	17.5	17	40	204.5	292.7
**20**	450	50	17.5	15	55	201.5	292.7
**21**	475	35	5	15	70	202.1	295.7
**22**	475	20	17.5	15	70	201.5	294.4
**23**	475	50	17.5	15	40	204.3	298.5
**24**	475	35	5	15	40	202.1	294.3
**25**	450	35	17.5	15	70	196.4	292.4
**26**	450	35	17.5	13	55	199.9	295.5
**27**	475	35	17.5	15	55	207.1	297.5
**28**	450	35	17.5	17	55	198.8	291.3
**29**	475	35	17.5	13	40	205.7	297.1
**30**	500	35	30	15	55	206.5	298.0
**31**	475	35	30	17	55	202.3	291.7
**32**	475	35	17.5	17	70	203.1	294.8
**33**	500	35	17.5	17	55	204.3	297.6
**34**	500	50	17.5	15	55	208.5	299.7
**35**	475	50	30	15	55	204.3	297.7
**36**	475	35	30	13	55	202.9	293.2
**37**	475	35	30	15	70	204.4	296.9
**38**	475	35	17.5	15	55	205.1	297.9
**39**	475	35	17.5	15	55	205.5	298.4
**40**	475	35	30	15	40	204.1	291.6
**41**	475	20	30	15	55	203.9	294.0
**42**	450	20	17.5	15	55	198.5	286.1
**43**	475	20	17.5	13	55	202.3	295.1
**44**	500	35	17.5	15	70	203.5	296.8
**45**	475	50	5	15	55	204.8	296.3
**46**	475	35	5	17	55	198.7	296.1

**Fig 6:**
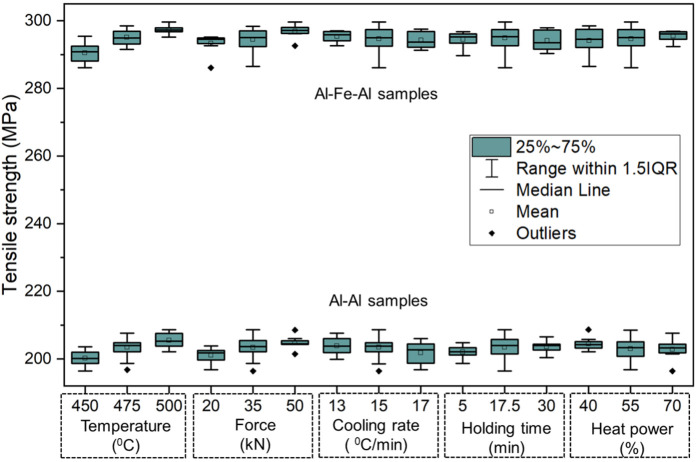
Distribution of tensile strength values for Al–Al and Al–Fe–Al samples at different levels of the processing parameters.

Fig 6 presents a boxplot-based overview of the experimental tensile strength data obtained from the Box–Behnken design. The figure is intended to visualize the distribution of the measured responses at different levels of each processing parameter before detailed statistical modeling. In general, the Al–Fe–Al laminated samples exhibit higher tensile strength than the Al–Al bonded samples under the investigated processing conditions. This result is reasonable because the steel core in the Al–Fe–Al structure contributes to the load-bearing capacity of the laminated specimen.

For both Al–Al and Al–Fe–Al samples, the tensile strength tends to increase when the pressing temperature increases from 450 to 500°C. This trend suggests that higher bonding temperature promotes interfacial bonding by increasing atomic mobility and improving plastic deformation at the contact interface. Pressing force also shows a positive influence on tensile strength, particularly when the force increases from 20 to 50 kN. Higher pressure can increase the real contact area between the stacked layers, reduce interfacial voids, and promote stronger bonding during hot pressing.

The effect of cooling rate is less straightforward. For the Al–Al samples, the tensile strength shows a slight decreasing tendency at the higher cooling rate, while the Al–Fe–Al samples show a relatively small variation across the investigated cooling-rate range. This indicates that cooling rate may have a secondary influence compared with pressing temperature and pressing force. Rapid cooling may contribute to residual stress or limit post-bonding atomic rearrangement, but its effect is not as dominant as the thermal and pressure conditions during bonding.

For holding time and heating power, no clear monotonic trend can be observed from the boxplot. The tensile strength values at different levels of these parameters partially overlap, indicating that their direct effects are relatively limited within the selected experimental range. Their influence may be associated with nonlinear behavior or interaction effects with other parameters rather than a strong independent linear effect. A few outlier points are observed in some parameter groups; however, these points were retained in the analysis because they may reflect the combined effects of multiple processing parameters in the BBD matrix rather than simple measurement errors.

Overall, the boxplot provides a useful preliminary visualization of the experimental dataset. It suggests that pressing temperature and pressing force are the most influential parameters affecting tensile strength, while cooling rate has a secondary effect and holding time and heating power show less obvious direct influence. These findings provide a solid foundation for subsequent optimization using the developed RSM model and NSGA-II algorithm.

### Box–Behnken Design (BBD) and Analysis of Variance (ANOVA)

Box–Behnken Design (BBD) is a Response Surface Methodology (RSM) technique used to develop second-order regression models that describe the relationship between input variables and output responses. Proposed by George E. P. Box and Donald Behnken (1960) [59], this method aims to reduce the number of required experimental runs while ensuring the accurate modeling of linear, quadratic, and interaction effects. In this study, BBD was employed to investigate the influence of five hot-pressing parameters—pressing temperature (A), pressing force (B), holding time (C), cooling rate (D), and heating power (E)—on the tensile strength of Al–Al and Al–Fe–Al laminated structures. To facilitate model construction and statistical analysis, the variables were coded into dimensionless forms at three levels: low (−1), center (0), and high (+1). Unlike full factorial designs, BBD positions experimental points at the midpoints of the edges of the design space and at the center, excluding the vertices of the design space. Consequently, this method avoids extreme experimental conditions that could be unstable, hazardous, or difficult to execute in practice. This represents a significant advantage in material processing, particularly in hot-press diffusion bonding, where parameters such as temperature and pressure are subject to strict operating limits.

For a system with k independent variables, the total number of experimental runs in a BBD is determined by the following formula:


$$\begin{array}{c} N=\text{2}k(k-\text{1})+C_{\text{0}} \end{array}$$ (1)

where C₀ is the number of center-point replicates, which are used to estimate pure experimental error and verify the model’s goodness-of-fit. In this study, k  =  5; therefore, 40 non-center design points were required. In addition, six replicated center points were included to evaluate experimental repeatability and estimate pure error. As a result, the total number of experimental runs was 46. The replicated center points corresponded to the middle levels of all five factors: pressing temperature of 475 °C, pressing force of 35 kN, holding time of 17.5 min, cooling rate of 15 °C/min, and heating power of 55%.

The obtained experimental data were utilized to construct the general second-order regression equation as follows:


$$\begin{array}{c} Y=a_{\text{0}}+\sum_{i=\text{1}}^{k}a_{i}x_{i}+\sum_{i=\text{1}}^{k}a_{ii}x_{i}^{\text{2}}+\sum_{\begin{array}{c} i\neq j\\ i,j=\text{1} \end{array}}^{k}a_{ij}x_{i}x_{j} \end{array}$$ (2)

In this equation, Y is the output response; a₀ is a constant; aᵢ, aᵢᵢ, and aᵢⱼ are the linear, quadratic, and interaction regression coefficients, respectively; and xᵢ and xⱼ are the coded variables. This model allows the evaluation of individual factor effects as well as their interactions on tensile strength.

The model’s goodness-of-fit was evaluated through Analysis of Variance (ANOVA), including the F-value, p-value, coefficient of determination (R^2^), adjusted R^2^, predicted R^2^, residual analysis, and lack-of-fit test. A model is generally considered statistically significant when its p-value is less than 0.05, while a non-significant lack-of-fit result indicates that the model adequately represents the experimental data within the investigated range. In addition, predicted-versus-experimental plots was used to further evaluate the adequacy of the regression models.

Table 4 shows the ANOVA results for the tensile strength of Al–Al samples. The model is statistically significant, with an F-value of 3.16 and a p-value of 0.004 (< 0.05). This confirms that the selected processing parameters collectively have a meaningful influence on the tensile strength of the Al–Al bonded specimens. Furthermore, the lack-of-fit test is not significant (p = 0.834), suggesting that the regression model adequately represents the experimental data within the investigated processing range. Among the linear terms, pressing temperature (A) and pressing force (B) are identified as the most significant factors, with very low p-values of 0.000 and 0.001, respectively. The high F-values for these parameters further indicate their dominant contribution to tensile strength, confirming the trends observed in the preliminary analysis. Physically, higher temperature enhances atomic mobility and promotes interfacial bonding, while increased pressure improves the real contact area between the mating surfaces and reduces void formation, leading to stronger bonding.

**Table 4:** ANOVA of tensile strength of Al-Al (BBD).

Source	DF	Adj SS	Adj MS	F-Value	P-Value
**Model**	20	258.783	12.939	3.16	0.004
**Linear**	5	203.802	40.760	9.95	0.000
**A (Temperature)**	1	112.360	112.360	27.44	0.000
**B (Force)**	1	54.760	54.760	13.37	0.001
**C (Holding time)**	1	9.201	9.201	2.25	0.146
**D (Cooling rate)**	1	16.810	16.810	4.11	0.054
**E (Heating power)**	1	10.671	10.671	2.61	0.119
**Square**	5	26.443	5.289	1.29	0.299
**A²**	1	10.295	10.295	2.51	0.125
**B²**	1	8.296	8.296	2.03	0.167
**C²**	1	10.507	10.507	2.57	0.122
**D²**	1	10.937	10.937	2.67	0.115
**E²**	1	0.000	0.000	0.00	0.997
**2-Way Interaction**	10	28.538	2.854	0.70	0.718
**A × B**	1	3.004	3.004	0.73	0.400
**A × C**	1	5.138	5.138	1.25	0.273
**A × D**	1	0.218	0.218	0.05	0.819
**A × E**	1	1.000	1.000	0.24	0.625
**B × C**	1	3.240	3.240	0.79	0.382
**B × D**	1	11.560	11.560	2.82	0.105
**B × E**	1	0.444	0.444	0.11	0.745
**C × D**	1	1.138	1.138	0.28	0.603
**C × E**	1	0.018	0.018	0.00	0.948
**D × E**	1	2.778	2.778	0.68	0.418
**Error**	25	102.363	4.095	—	—
**Lack-of-Fit**	20	71.047	3.552	0.57	0.834
**Pure Error**	5	31.316	6.263	—	—
**Total**	45	361.146	—	—	—

In contrast, cooling rate (D) shows a marginal effect with a p-value of 0.054, indicating a secondary influence that may still be relevant near the significance threshold. Meanwhile, holding time (C) and heating power (E) exhibit relatively high p-values of 0.146 and 0.119, respectively, suggesting that their direct linear effects on tensile strength are not statistically significant within the investigated range. For higher-order terms, the quadratic effects are generally not significant, indicating that curvature effects are relatively weak within the studied parameter range. Similarly, the two-way interaction terms show no significant influence overall, although the B × D interaction presents a relatively lower p-value, implying a possible but limited interaction between pressing force and cooling rate.

The resulting regression equations for the tensile strength of Al–Al samples are presented as follows:


$$\begin{array}{cc} \sigma (\text{Al}-\text{Al})= & -\text{220}+\text{1}.\text{61A}-\text{1}.\text{52B}-\text{1}.\text{59C}+\text{9}.\text{3D}-\text{0}.\text{33E}-\text{0}.{\text{00174A}}^{2}-\text{0}.{\text{00433B}}^{2}-\text{0}.{\text{00702C}}^{2}-\\ & \text{0}.{\text{280D}}^{2}+\text{0}.\text{00001 }{\text{E}}^{2}+\text{0}.\text{00231 A}*\text{B}+\text{0}.\text{00363A}*\text{C}-\text{0}.\text{0047A}*\text{D}+\text{0}.\text{00133A}*\text{E}-\text{0}.\text{00480B}*\text{C}\\ & +\text{0}.\text{0567B}*\text{D}+\text{0}.\text{00148B}*\text{E}+\text{0}.\text{0213C}*\text{D}+\text{0}.\text{00036C}*\text{E}-\text{0}.\text{0278D}*\text{E} \end{array}$$ (3)

**Fig 7:**
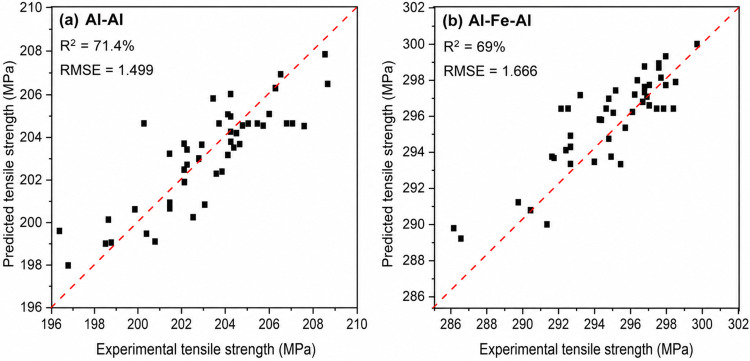
Comparison between experimental and predicted tensile strength values obtained from the RSM regression models for (a) Al–Al and (b) Al–Fe–Al samples.

The coefficient of determination of the Al–Al model is R^2^ = 71.66%, indicating that approximately 71.66% of the variation in tensile strength can be explained by the developed regression model. Although the adjusted R^2^ value decreases to 48.98% because the full quadratic model contains a relatively large number of higher-order and interaction terms compared with the number of experimental runs, the predictive capability of the model was further evaluated using an independent predicted-versus-experimental parity plot in Fig 7(a). The parity plot shows that the predicted values generally follow the experimental trend, with a correlation coefficient of R² = 0.714 and a relatively low root mean square error of RMSE = 1.499 MPa. These results indicate that the model does not show serious overfitting and can provide reasonably stable prediction within the investigated design space. Therefore, although the model should still be interpreted with caution, it is considered suitable for identifying dominant processing parameters, analyzing process trends, and supporting optimization within the selected parameter ranges. However, the model should not be extrapolated beyond the investigated design space.

Table 5 presents the ANOVA results for the tensile strength of Al–Fe–Al samples. The developed model is statistically significant, with a p-value of 0.001, and the lack-of-fit test is not significant (p = 0.928), indicating good agreement between the model and the experimental data. Similar to the Al–Al case, the linear terms dominate the model, with pressing temperature (A) showing the strongest influence, as indicated by an F-value of 45.24 and a p-value of 0.000. Pressing force (B) is also significant, with a p-value of 0.002. This highlights the critical role of thermal activation and mechanical pressure in enhancing bonding performance in the Al–Fe–Al laminated structure.

**Table 5:** ANOVA of tensile strength of Al- Fe – Al (BBD).

Source	DF	Adj SS	Adj MS	F-Value	P-Value
**Model**	20	308.144	15.407	3.75	0.001
**Linear**	5	249.851	49.970	12.16	0.000
**A (Temperature)**	1	185.868	185.868	45.24	0.000
**B (Force)**	1	50.884	50.884	12.38	0.002
**C (Holding time)**	1	0.490	0.490	0.12	0.733
**D (Cooling rate)**	1	5.138	5.138	1.25	0.274
**E (Heating power)**	1	7.471	7.471	1.82	0.190
**Square**	5	22.981	4.596	1.12	0.376
**A²**	1	15.194	15.194	3.70	0.066
**B²**	1	0.634	0.634	0.15	0.698
**C²**	1	5.702	5.702	1.39	0.250
**D²**	1	0.149	0.149	0.04	0.851
**E²**	1	0.149	0.149	0.04	0.851
**2-Way Interaction**	10	35.311	3.531	0.86	0.580
**A × B**	1	1.000	1.000	0.24	0.626
**A × C**	1	0.071	0.071	0.02	0.896
**A × D**	1	5.444	5.444	1.33	0.261
**A × E**	1	12.484	12.484	3.04	0.094
**B × C**	1	1.284	1.284	0.31	0.581
**B × D**	1	3.240	3.240	0.79	0.383
**B × E**	1	0.360	0.360	0.09	0.770
**C × D**	1	6.084	6.084	1.48	0.235
**C × E**	1	3.738	3.738	0.91	0.349
**D × E**	1	1.604	1.604	0.39	0.538
**Error**	25	102.723	4.109	—	—
**Lack-of-Fit**	20	64.015	3.201	0.41	0.928
**Pure Error**	5	38.708	7.742	—	—
**Total**	45	410.866	—	—	—

In contrast, holding time (C), cooling rate (D), and heating power (E) exhibit high p-values, indicating negligible direct effects on tensile strength within the investigated range. The quadratic and interaction terms are also statistically insignificant overall, although A^2^ and A × E show relatively lower p-values near the significance threshold. These terms may indicate weak nonlinear or interaction tendencies.

For the Al–Fe–Al model, the coefficient of determination reaches R^2^ = 75.00%, showing that the regression model can explain approximately three-quarters of the variation in tensile strength. The adjusted R^2^ value decreases to 55.00%, which is expected because the full quadratic model includes multiple linear, quadratic, and interaction terms relative to the number of experimental observations. To further assess the practical predictive performance of the model, a predicted-versus-experimental parity plot was constructed in Fig 7(b). The parity plot indicates that the predicted tensile strength values follow the overall experimental tendency, with a correlation coefficient of R² = 0.690 and an RMSE of 1.666 MPa. Although some deviations from the ideal prediction line are observed, the relatively low RMSE suggests that the model can still provide acceptable prediction accuracy within the studied parameter range. Therefore, the Al–Fe–Al model is considered adequate for describing the main process–response relationship and supporting the subsequent optimization step. Nevertheless, due to the moderate adjusted R² value, the model should be used only within the investigated design space and should not be extrapolated beyond the selected processing conditions.

The resulting regression equations for the tensile strength of Al–Fe–Al samples are presented as follows:


$$\begin{array}{cc} \sigma (\text{Al}-\text{Fe}-\text{Al})= & -\text{208}+\text{2}.\text{09A}+\text{0}.\text{24B}+\text{0}.\text{32C}-\text{11}.\text{7D}+\text{1}.\text{99E}-\text{0}.{\text{00211A}}^{2}\\ & +\text{0}.{\text{00120B}}^{2}-\text{0}.{\text{00517C}}^{2}-\text{0}.{\text{033D}}^{2}-\text{0}.{\text{00058E}}^{2}-\text{0}.\text{00133A}*\text{B}\\ & +\text{0}.\text{00043A}*\text{C}+\text{0}.\text{0233A}*\text{D}-\text{0}.\text{00471A}*\text{E}+\text{0}.\text{00302B}*\text{C}\\ & +\text{0}.\text{0300B}*\text{D}-\text{0}.\text{00133B}*\text{E}-\text{0}.\text{0493C}*\text{D}+\text{0}.\text{00516C}*\text{E}+\text{0}.\text{0211D}*\text{E} \end{array}$$ (4)

Overall, the ANOVA results confirm that pressing temperature and pressing force are the dominant factors governing the tensile strength of both Al–Al and Al–Fe–Al bonded structures. Cooling rate has a marginal or secondary effect, while holding time and heating power show limited direct influence within the selected processing range. These findings provide the statistical basis for the subsequent NSGA-II multi-objective optimization.

### NSGA-II multi-objective optimization

Although only tensile strength is considered as the mechanical performance indicator, the presence of two different material systems, Al–Al and Al–Fe–Al, makes this a multi-objective problem. This is because the processing conditions that maximize the tensile strength of the Al–Al joint may not necessarily provide the maximum tensile strength of the Al–Fe–Al laminated structure. Therefore, NSGA-II was applied to optimize both tensile strength responses simultaneously and to identify the Pareto front. The knee point was selected as the best compromise solution, providing balanced performance for both material systems.

After constructing the second-order regression models using Response Surface Methodology, the multi-objective optimization problem was solved using the Non-dominated Sorting Genetic Algorithm II (NSGA-II). The objective functions used in the optimization were based on the RSM regression models for the tensile strength of Al–Al and Al–Fe–Al samples. The optimization problem can be expressed as follows:


$$\begin{array}{c} \text{Maximize }{\text{f}}_{\text{1}}(\text{X})\;=\;\sigma \text{t}(\text{Al}-\text{Al}) \end{array}$$ (5)

$$\begin{array}{c} \text{Maximize }{\text{f}}_{\text{2}}(\text{X})\;=\;\sigma \text{t}(\text{Al}-\text{Fe}-\text{Al}) \end{array}$$ (6)

where X = [A, B, C, D, E] represents the vector of processing parameters, including pressing temperature, pressing force, holding time, cooling rate, and heating power. The optimization was constrained within the same parameter ranges used in the Box–Behnken design: 450 ≤ A ≤ 500 °C, 20 ≤ B ≤ 50 kN, 5 ≤ C ≤ 30 min, 13 ≤ D ≤ 17 °C/min, and 40 ≤ E ≤ 70%. These bounds were used to ensure that the optimization was performed only within the experimentally investigated design space.

The NSGA-II algorithm is an efficient evolutionary method proposed by Kalyanmoy Deb and has been widely applied in multi-objective optimization problems due to its ability to identify a set of Pareto-optimal solutions [60]. In this study, the algorithm was used to determine the optimal combination of hot-press diffusion bonding parameters for improving the tensile strength of both bonded structures. The main parameters of the NSGA-II algorithm were set as follows: the population size was 200 individuals, the number of generations was 1000, the crossover probability was 0.9, and polynomial mutation was used to maintain population diversity. The selection process was carried out based on non-dominated sorting combined with the crowding distance metric, which prioritizes solutions with higher Pareto ranks and ensures a well-distributed set of solutions in the search space.

**Fig 8:**
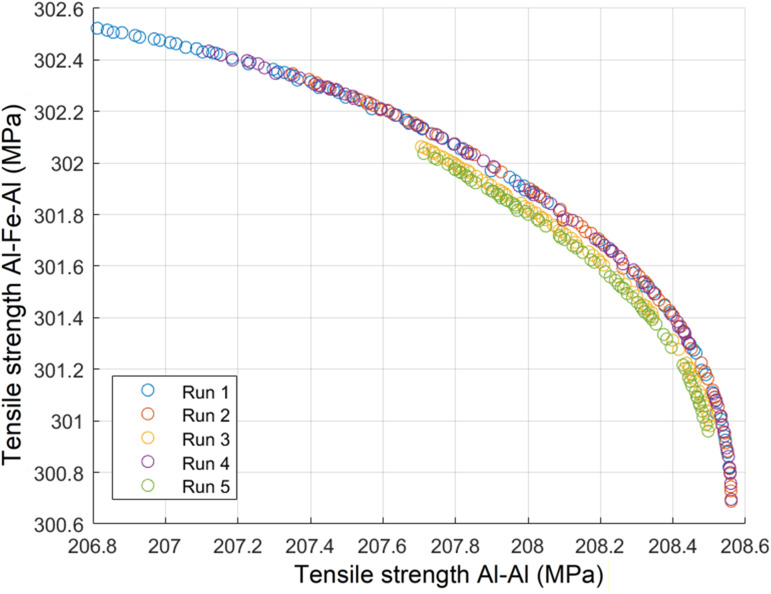
Comparison of Pareto fronts obtained from five independent NSGA-II runs.

The stopping criterion of the algorithm was defined as reaching the maximum number of generations. To improve the reliability and repeatability of the optimization results, the NSGA-II calculation was repeated five independent times using the same objective functions, constraints, and algorithmic settings. The objective functions in this study were constructed based on the RSM regression models, enabling rapid evaluation of response values without the need to conduct additional experiments for each optimization candidate. Therefore, the optimization results should be interpreted within the investigated experimental range. The Pareto fronts obtained from the five independent runs are compared in Fig 8.

As shown in Fig 8, the Pareto fronts obtained from the five independent NSGA-II runs exhibit a highly similar distribution and follow the same trade-off trend between the tensile strength of the Al–Al and Al–Fe–Al structures. This result indicates that the optimization process was stable and that the obtained non-dominated solutions were not strongly affected by random initialization. The small spread among the fronts is expected because NSGA-II is a stochastic evolutionary algorithm. Nevertheless, the close overlap of most solutions confirms good convergence consistency. The obtained Pareto fronts also show a clear trade-off relationship between the two objectives: solutions with higher predicted tensile strength for the Al–Al structure tend to be associated with slightly lower predicted tensile strength for the Al–Fe–Al laminated structure. Therefore, instead of selecting an extreme solution that favors only one material system, a knee-point solution was selected to provide a balanced compromise between the two objectives.

**Fig 9:**
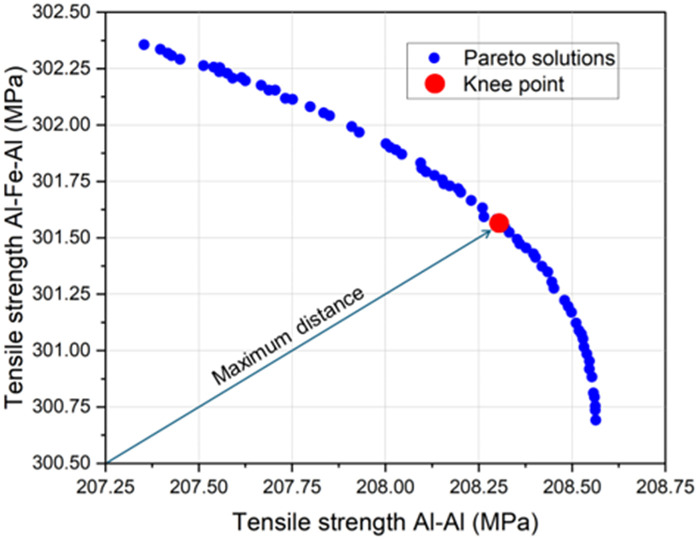
Pareto front and knee point.

The obtained Pareto set was used to identify the knee point, defined as the solution with the maximum perpendicular distance from the line connecting the two extreme solutions. This knee point represents the most balanced solution between the two tensile strength responses. The Pareto front and knee point identified from the independent NSGA-II runs are shown in Fig 9. The knee point occurs at A ≈ 500 °C, B = 50 kN, C ≈ 18.87 min, D ≈ 16.50 °C/min, and E ≈ 40%, yielding predicted tensile strengths of σt(Al–Al) = 208.30 MPa and σt(Al–Fe–Al) = 301.58 MPa.

This parameter set enables the two-material system to achieve near-optimal and well-balanced tensile performance. Nearby Pareto solutions exhibit very similar parameter values and only slight variations in tensile strength, confirming that this region is relatively stable and governs the optimal behavior of the material system. Furthermore, moving away from the knee point along the Pareto front results in a trade-off, where improving one response may reduce the other. Thus, the knee point represents the most practical compromise solution for the dual-material bonding problem.

To verify the reliability of the NSGA-II predicted optimum, confirmation experiments were conducted using the optimized processing parameters obtained from the knee-point solution. The selected confirmation condition was a pressing temperature of 500 °C, pressing force of 50 kN, holding time of 18.87 min, cooling rate of 16.50 °C/min, and heating power of 40%. The experimentally measured tensile strengths were then compared with the predicted values obtained from the RSM–NSGA-II optimization model. The confirmation results are summarized in Table 6.

**Table 6:** Confirmation experiment results for optimal processing parameters.

Response	Predicted value (MPa)	Experimental value (MPa)	Deviation (%)
**σt(Al–Al)**	208.30	208.50	0.10
**σt(Al–Fe–Al)**	301.58	299.70	0.62

The confirmation results show good agreement between the predicted and experimental tensile strength values. For the Al–Al bonded structure, the deviation between the predicted value of 208.30 MPa and the experimental value of 208.50 MPa was only 0.10%. For the Al–Fe–Al laminated structure, the predicted value was 301.58 MPa, while the experimental value was 299.70 MPa, corresponding to a deviation of 0.62%. These low deviation values indicate that the RSM–NSGA-II optimization model can reasonably predict the tensile strength responses within the investigated design space. Therefore, the selected knee-point solution can be considered a reliable and practical processing condition for achieving balanced tensile performance in both Al–Al and Al–Fe–Al bonded structures.

### SEM image analysis

**Fig 10:**
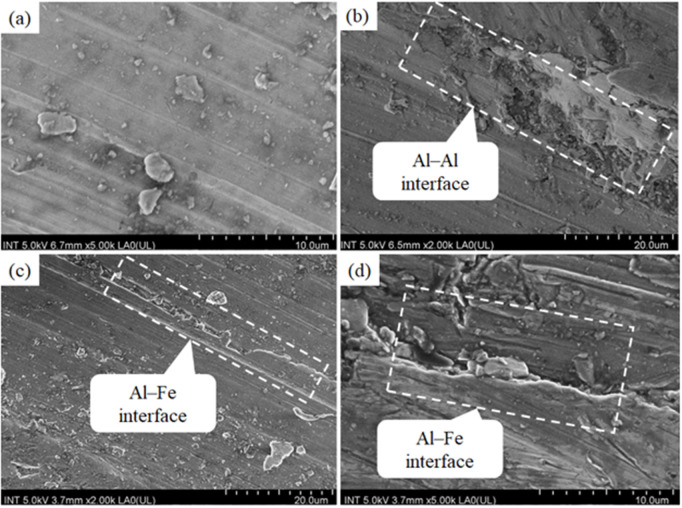
SEM images of the bonded interfaces before and after tensile testing. SEM images of the Al–Al interface before tensile testing (a) and after tensile testing (b), and the Al–Fe interface in the Al–Fe–Al laminated structure before tensile testing (c) and after tensile testing (d).

Fig 10 illustrate the interfacial characteristics and fracture features of Al–Al and Al–Fe–Al samples before and after tensile testing. The samples exhibiting optimum or near-optimum tensile strength were selected for SEM analysis in order to qualitatively examine the interfacial morphology and fracture behavior associated with improved mechanical performance. It should be noted that SEM analysis in this study was used mainly to observe interfacial continuity, crack formation, and fracture morphology. Phase identification and elemental diffusion analysis were not performed; therefore, the formation, distribution, and thickness of possible Fe–Al intermetallic compounds were not directly confirmed in the present work.

In the Al–Al sample, the interface within the gauge length before testing, as shown in Fig 10(a), is difficult to distinguish clearly. This indicates relatively good interfacial contact between the two aluminum layers after hot-press diffusion bonding. This behavior can be attributed to the similarity of the two aluminum surfaces, plastic deformation of surface asperities under compressive force, and improved contact promoted by elevated bonding temperature. After tensile testing, as shown in Fig 10(b), localized damage and micro-cracks are observed near the interfacial region. However, complete separation along the original bonding interface is not clearly observed, suggesting that the Al–Al interface maintained relatively good bonding integrity during tensile loading. The observed micro-cracks may have initiated from local stress concentration or small interfacial defects remaining after bonding.

In contrast, the Al–Fe–Al sample shows a clearly defined interface before tensile testing, as shown in Fig 10(c). This indicates that the interface between aluminum and steel remained more distinguishable after bonding because of the dissimilar nature of the two materials. Compared with the Al–Al interface, the Al–Fe interface is more difficult to bond uniformly because aluminum and steel have different crystal structures, mechanical properties, thermal expansion coefficients, and diffusion behavior. In addition, the stable oxide film on aluminum and the limited mutual solubility between Al and Fe can hinder the formation of a fully continuous metallurgical bond.

After tensile testing, as shown in Fig 10(d), significant interfacial separation and damage are observed in the Al–Fe–Al specimen. The fracture path tends to occur along or near the Al–Fe interface, indicating that this region is the weakest part of the laminated structure under tensile loading. This behavior can be explained by the combined effects of weaker interfacial compatibility, mechanical mismatch between aluminum and steel, and local stress concentration at the dissimilar-material interface. During tensile deformation, the difference in elastic modulus between aluminum and steel can lead to strain incompatibility, while the difference in thermal expansion coefficient may also contribute to residual stress after cooling. These effects can promote local interfacial shear stress and accelerate debonding along the Al–Fe interface.

Although Fe–Al intermetallic compounds such as Fe_2_Al₅ and FeAl_3_ are commonly reported in Al–steel bonding systems, their presence in the present specimens cannot be confirmed without phase or elemental analysis. Therefore, the discussion of possible interfacial reactions is limited to a literature-based explanation and is not used as direct experimental evidence in this study. The SEM results mainly demonstrate the difference in fracture behavior between similar Al–Al bonding and dissimilar Al–Fe–Al laminated bonding. The Al–Al sample shows a more continuous interface and localized crack propagation, whereas the Al–Fe–Al sample shows more obvious interfacial separation after tensile loading.

Overall, the SEM observations support the tensile test results by showing that the Al–Al joint has better interfacial continuity, while the Al–Fe–Al laminated structure is more sensitive to interfacial debonding. These results suggest that pressing temperature and pressing force improve bonding performance by promoting contact and deformation at the interface, but the Al–Fe–Al structure remains more challenging because of the mechanical and physical mismatch between aluminum and steel. A limitation of the present study is that EDS, XRD, TEM, or diffusion-layer thickness measurements were not available. Therefore, future work should include elemental mapping, phase identification, and quantitative interfacial layer measurement to clarify the detailed bonding mechanism of Al–Fe–Al laminated structures.

## Conclusions

This study investigated the hot-press diffusion bonding of Al–Al and Al–Fe–Al laminated structures using a Box–Behnken Design, Response Surface Methodology, and NSGA-II multi-objective optimization. The effects of pressing temperature, pressing force, holding time, cooling rate, and heating power on tensile strength were analyzed, and the following conclusions can be drawn:

The BBD–RSM approach was successfully used to model the relationship between hot-press diffusion bonding parameters and tensile strength for both Al–Al and Al–Fe–Al bonded structures. The ANOVA results showed that the regression models were statistically significant for both responses, with p-values lower than 0.05 and non-significant lack-of-fit values. This indicates that the developed models can adequately describe the experimental results within the investigated processing range.

Pressing temperature and pressing force were identified as the dominant parameters affecting tensile strength in both material systems. For the Al–Al samples, pressing temperature and pressing force showed the strongest statistical significance, while cooling rate had a marginal secondary effect. For the Al–Fe–Al laminated samples, pressing temperature was the most influential factor, followed by pressing force. Holding time and heating power showed limited direct influence within the selected parameter ranges.

The NSGA-II multi-objective optimization identified a knee-point solution that provides a balanced improvement in tensile strength for both bonded structures. The optimized processing condition was approximately 500 °C pressing temperature, 50 kN pressing force, 18.87 min holding time, 16.50 °C/min cooling rate, and 40% heating power. Under this condition, the predicted tensile strengths were 208.30 MPa for the Al–Al bonded structure and 301.58 MPa for the Al–Fe–Al laminated structure. The confirmation experiment showed good agreement with the predicted results, giving experimental tensile strengths of 208.50 MPa for Al–Al and 299.70 MPa for Al–Fe–Al, with deviations of only 0.10% and 0.62%, respectively. These results confirm that the selected knee-point solution is a reliable processing condition within the investigated design space.

The optimization results indicate that high pressing temperature and high pressing force are beneficial for improving tensile performance because they promote better interfacial contact, plastic deformation of surface asperities, and bonding development during hot pressing. The selected intermediate holding time suggests that excessive holding time is not necessary once sufficient temperature and pressure are applied within the investigated range.

SEM observations provided qualitative evidence of different fracture behaviors in the two bonded systems. The Al–Al specimens showed a relatively continuous interface and localized micro-crack formation after tensile loading, suggesting better interfacial continuity. In contrast, the Al–Fe–Al specimens showed a clearer dissimilar-material interface and more obvious interfacial separation after tensile testing, indicating that the Al–Fe interface remains the more critical region in the laminated structure.

The main contribution of this work is the application of an integrated experimental–statistical–optimization framework to simultaneously evaluate and optimize the tensile performance of similar Al–Al bonding and dissimilar Al–Fe–Al laminated bonding under the same hot-press diffusion bonding conditions. This provides practical guidance for selecting processing parameters for aluminum-based laminated structures.

This study also has limitations. First, the evaluation of bonding performance was mainly based on tensile strength, while other mechanical properties such as shear strength, fatigue behavior, hardness distribution, and residual stress were not investigated. Second, SEM observation was used only for qualitative interfacial and fracture analysis. Phase identification and elemental diffusion analysis using EDS, XRD, or TEM were not performed; therefore, the formation, distribution, and thickness of possible Fe–Al intermetallic compounds could not be directly confirmed. Third, the NSGA-II optimum was obtained based on RSM predictive models within the investigated design space.

Future work should therefore include quantitative interfacial characterization, elemental mapping, phase identification, diffusion-layer thickness measurement, and broader mechanical testing. These additional analyses would provide a deeper understanding of the bonding mechanism and further improve the reliability of process optimization for Al–Fe–Al laminated structures.

## Supporting information

S1 File MATLAB codes, multi-run results, knee-point analysis and figures for NSGA-II multi-objective optimization of diffusion bonding parameters (Al–Al and Al–Fe–Al). This supporting file contains the complete MATLAB implementation and results of the NSGA-II optimization used to maximize the two tensile strengths σt (Al–Al) and σt (Al–Fe–Al) simultaneously. The archive includes the objective function, optimization scripts with the exact algorithm settings, five independent runs, knee-point selection, representative Pareto solutions, and corresponding figures.
(ZIP)
