# Supplementary material for: Optimization of process parameters for enhancing the tensile strength of diffusion-bonded Al–Al and Al–Fe–Al laminated structures
Source: PLoS One. 2026 Aug 11;21(8):e0354878. doi: 10.1371/journal.pone.0354878 (PMC13460570; doi:10.1371/journal.pone.0354878)
Supplement: Supplementary file 1 [file pone.0354878.s003.docx]

Dear Reviewer #2,

I hope this message finds you well.

On behalf of the entire authorship team, I would like to express our sincere gratitude for your careful and constructive review of our manuscript. Your detailed comments and valuable suggestions have substantially improved the clarity, technical quality, and overall presentation of the paper.

We have thoroughly addressed all of your points in the revised version. The major revisions include:

*1. Abstract is not well drafted. Write a structured abstract mentioning the statistical data, but within the journal’s template. It is advised to follow IMRAD template for Headings in the article.*

**Respond:**

Thank you for this helpful comment. The Abstract has been revised to improve its clarity, structure, and focus. The revised Abstract now follows a clearer background–gap–method–result–contribution flow and better reflects the main content of the manuscript. It emphasizes the hot-press diffusion bonding process, dual-material optimization of Al–Al and Al–Fe–Al structures, BBD–RSM modeling, NSGA-II optimization, and tensile performance. In addition, the manuscript has been reformatted according to the journal template, including the use of appropriate heading levels. The revised Abstract also avoids overclaiming detailed diffusion-mechanism analysis because EDS/XRD and diffusion-layer thickness measurements were not performed in the present study.

*2. The abstract claims “limited research on Al-Fe-Al laminated structures” but fails to quantify how many prior studies exist. Please specify the gap with concrete citations.*

**Respond:**

Thank you for this comment. The Abstract and Introduction have been revised to clarify the research gap more carefully and to support the statement with more specific literature discussion. Instead of making a broad unsupported claim, the revised manuscript now explains that most previous studies have focused on direct Al–Fe interfaces, interlayer-assisted Al–Fe bonding, or friction-based Al–Fe joining, whereas fewer studies have addressed Al–Fe–Al laminated structures in which steel serves as the core layer and aluminum acts as the outer layers (No research has investigated this issue). Recent studies were added to strengthen this point and to show that the present work specifically investigates Al–Fe–Al laminated structures and the simultaneous optimization of Al–Al and Al–Fe–Al systems under the same hot-press diffusion bonding framework.

The correction has been made in Page 1 and Introduction part.

*3. NSGA-II optimization is mentioned, yet the abstract does not state whether the predicted optima were experimentally validated. This must be clarified.*

**Respond:**

Thank you for this important comment. We have clarified this issue in the revised manuscript. The NSGA-II predicted optimum was experimentally verified, and the validation results have now been added on page 25 of the revised manuscript, immediately after the Pareto front and knee-point optimization results. Specifically, confirmation experiments were conducted using the optimized processing parameters obtained from the knee-point solution: pressing temperature of 500 °C, pressing force of 50 kN, holding time of 18.87 min, cooling rate of 16.50 °C/min, and heating power of 40%.

The predicted tensile strengths obtained from the RSM–NSGA-II model were 208.30 MPa for the Al–Al bonded structure and 301.58 MPa for the Al–Fe–Al laminated structure. The corresponding experimental values were 208.50 MPa and 299.70 MPa, respectively. The deviations between predicted and experimental values were only 0.10% for Al–Al and 0.62% for Al–Fe–Al. These low deviations confirm that the predicted optimum was experimentally validated and that the RSM–NSGA-II model can reasonably predict the tensile strength responses within the investigated design space.

*4. The introduction cites several review papers but does not highlight any specific contradictory findings from previous diffusion bonding studies. Add a clear statement of unresolved discrepancies.*

**Respond:**

Thank you for this valuable comment. The Introduction has been revised to include a more critical discussion of previous findings, particularly regarding Fe–Al intermetallic compounds in Al–Fe bonding systems. The revised manuscript now explains that some studies reported that thin or discontinuous intermetallic layers may not severely reduce bonding strength, whereas thick, continuous, or cracked intermetallic layers can significantly deteriorate interfacial performance. This unresolved discrepancy was added to highlight that the effect of Fe–Al intermetallic compounds depends not only on their presence, but also on their thickness, morphology, continuity, and crack formation. This discussion helps strengthen the motivation for investigating the process–property relationship in Al–Al and Al–Fe–Al laminated structures.

The correction has been made in Page 3.

*5. Cite more contemporary studies to strengthen the research gap. In this regards, the authors are encouraged to cite the following recent and highly relevant articles to strengthen the literature background. Incorporation the following works will improve the article’s readability:*

**Respond:**

Thank you for this valuable suggestion. We agree that incorporating more recent and relevant studies can strengthen the literature background and clarify the research gap of the present work. Following the reviewer’s recommendation, we have added and discussed the suggested contemporary references in the revised Introduction. These studies were integrated to update the background on aluminum-based materials, Al–Fe bonding, solid-state joining, interfacial behavior, and process optimization methods. Their inclusion helps position the present study more clearly within recent developments and highlights the need for a process-oriented investigation of Al–Al and Al–Fe–Al laminated structures under hot-press diffusion bonding conditions.

The correction has been made in Page 4.

*6. Also, it will be great if the authors add a summary of literature just after completion of prior state of the art.*

**Respond:**

Thank you for this suggestion. The Introduction has been reorganized to provide a clearer synthesis of previous studies after the state-of-the-art discussion. A summary-style gap paragraph has been added before the aim of the study. This paragraph identifies the main research gaps addressed in the present work, including the limited systematic investigation of Al–Al hot-press bonding, limited studies on Al–Fe–Al laminated structures, insufficient simultaneous optimization of both material systems, and the lack of direct connection between hot-press diffusion bonding parameters and dual tensile strength responses. This addition improves the logical transition from the literature review to the research objectives.

*7. The hole-pattern structure on the steel layer is described, but its geometry (diameter, depth, spacing) and how it affects mechanical interlocking are not quantified. Provide their details too.*

**Respond:**

Thank you for this helpful comment. The Materials section has been revised to describe the hole-pattern structure on the steel layer more clearly. The revised manuscript now provides the detailed geometry of the hole pattern, including the hole diameter, depth, spacing, number of holes, and patterned area fraction. In addition, the mechanical role of the pattern has been clarified. The hole pattern increases the effective contact area and promotes mechanical interlocking because softened aluminum can locally deform into the holes during hot pressing. The detailed dimensions of the hole pattern are also shown in Figure 2c.

*8. The tensile testing section follows ASTM E8/E8M, yet the reported specimen thickness (6 mm) and gauge length (57 mm) do not match standard subsize proportions. Verify and correct them.*

**Respond:**

Thank you for this important comment. We rechecked the specimen dimensions according to ASTM E8/E8M and corrected the description in the revised manuscript. In the previous version, 57 mm was incorrectly referred to as the gauge length. According to ASTM E8/E8M, 57 mm corresponds to the minimum reduced section length for the 12.5 mm wide sheet-type rectangular specimen, while the gauge length is 50 mm. The manuscript has been corrected accordingly. The specimen used in this study should be considered a 12.5 mm wide sheet-type rectangular specimen rather than a 6 mm wide subsize specimen. The reported thickness of 6 mm is within the allowable thickness range for the 12.5 mm wide sheet-type specimen.

The correction has been made in Page 12.

*9. Five processing parameters are studied, but the range for cooling rate (13–17°C/min) is extremely narrow. Justify why such a small window was chosen.*

**Respond:**

Thank you for this comment. The cooling-rate range has been justified in the revised experimental design and specimen processing sections. The revised manuscript explains that the cooling-rate window of 13–17 °C/min was selected based on the stable and controllable operating range of the hot-press system. This range was also chosen to avoid excessive thermal gradients, residual stress, and distortion during post-bonding cooling. Although the cooling-rate range is relatively narrow compared with the other factors, it was retained in the experimental design because cooling behavior can still influence the tensile performance of laminated structures, especially in dissimilar Al–Fe–Al systems.

The correction has been made in Page 9.

*10. Figure 5 plots use polynomial fitting without reporting R² or RMSE values. Add these goodness-of-fit metrics for each subplot.*

**Respond:**

Thank you for this helpful suggestion. The original polynomial fitting plots were replaced because they were not the most suitable way to present the Box–Behnken dataset, where multiple parameters change simultaneously. For better visualization of the experimental response data, a boxplot-based overview figure was added to show the distribution of tensile strength values at different parameter levels. In addition, the model adequacy discussion was strengthened by adding predicted-versus-experimental parity plots with R² and RMSE values for both Al–Al and Al–Fe–Al models. Therefore, the revised manuscript now presents the dataset overview and model prediction performance more clearly.

The correction has been made in Figure 6, 7.

*11. The discussion attributes weaker Al-Fe bonding to Young’s modulus mismatch, but no quantitative calculation of interfacial shear stress is provided. Include a simple estimate to support the claim.*

**Respond:**

Thank you for this important comment. The SEM discussion has been revised to avoid attributing the weaker Al–Fe interfacial bonding only to Young’s modulus mismatch. Instead, the revised manuscript explains the interfacial separation more cautiously as the combined result of weaker interfacial compatibility, mechanical mismatch between aluminum and steel, thermal expansion mismatch, local stress concentration, and possible residual stress after cooling. The discussion now also considers the effect of temperature and material mismatch on the Al–Fe interface. Because a quantitative interfacial shear stress calculation was not performed in the present study, the claim has been softened and presented as a qualitative interpretation supported by SEM observations and literature-based understanding.

The correction has been made in Page 25.

*12. The SEM images are presented without scale bars or magnification labels. Add these for proper interpretation of interfacial features.*

**Respond:**

Thank you for this comment. The SEM figure has been revised to include scale bars and magnification values for proper interpretation of the interfacial and fracture features. The figure caption has also been revised to indicate that scale bars and magnification values are shown in each SEM image. In addition, the manuscript text now clarifies that SEM observation was used only as qualitative evidence of interfacial morphology and fracture behavior, not for phase identification or diffusion-layer measurement.

The correction has been made in Figure 9.

*13. First of all, it must be “Conclusions”. In the current form, the presented conclusions section doesn’t provide any concrete outcome to the readers. It is suggested to include the limitations as well as the future work in the article.*

**Respond:**

Thank you for this helpful comment. The final section has been revised and renamed “Conclusions.” The revised Conclusions now provide more concrete outcomes, including the significant processing parameters identified by ANOVA, model significance, the optimized NSGA-II knee-point parameters, predicted tensile strength values, and SEM-based fracture observations. The limitations of the study have also been clearly added, including the absence of EDS/XRD/TEM analysis, diffusion-layer thickness measurement, broader mechanical testing, and additional confirmation experiments at the optimized condition. Future work has been added to include quantitative interfacial characterization, phase identification, hardness distribution, residual stress analysis, shear strength, fatigue behavior, and validation experiments.

The correction has been made in Page 26-28

Thank you once again for your time and insightful feedback, which were instrumental in improving our work.

Best regards,

**Pham Son Minh, PhD**

Corresponding Author Faculty of Mechanical Engineering

Ho Chi Minh City University of Technology and Education (HCMUTE)

Email: [minhps@hcmute.edu.vn](mailto:minhps@hcmute.edu.vn)
