# Supplementary material for: Optimization of process parameters for enhancing the tensile strength of diffusion-bonded Al–Al and Al–Fe–Al laminated structures
Source: PLoS One. 2026 Aug 11;21(8):e0354878. doi: 10.1371/journal.pone.0354878 (PMC13460570; doi:10.1371/journal.pone.0354878)
Supplement: Supplementary file 2 [file pone.0354878.s001.zip › NSGA-II-Matlab/NSGA-II.docx]

% NSGA-II options (paper settings)

opts = optimoptions('gamultiobj', ...

'PopulationSize', 200, ...

'MaxGenerations', 1000, ...

'CrossoverFraction', 0.9, ... % Pc = 0.9

'MutationFcn', {@mutationadaptfeasible, 0.25}, ... % Pm = 0.25

Để đánh giá độ ổn định và khả năng hội tụ của thuật toán NSGA-II, quá trình tối ưu đa mục tiêu đã được thực hiện lặp lại 5 lần với các giá trị seed ngẫu nhiên khác nhau. Các Pareto front thu được từ các lần chạy được thể hiện trong Hình X. Kết quả cho thấy các đường Pareto có hình dạng và xu hướng phân bố gần như trùng khớp, thể hiện mối quan hệ đánh đổi rõ ràng giữa hai mục tiêu cần tối ưu. Sự tương đồng này chứng tỏ thuật toán có khả năng hội tụ ổn định về cùng một vùng nghiệm tối ưu, bất chấp tính ngẫu nhiên vốn có của các toán tử di truyền.

Mặc dù tồn tại một số sai khác nhỏ về mật độ phân bố điểm nghiệm giữa các lần chạy, đặc biệt tại các vùng biên của Pareto front, các nghiệm ở vùng trung tâm – đại diện cho sự cân bằng tối ưu giữa hai mục tiêu – có mức độ chồng lấn cao. Điều này cho thấy các nghiệm Pareto thu được có tính nhất quán và độ tin cậy cao. Các sai khác nhỏ quan sát được có thể được giải thích bởi cơ chế lai ghép và đột biến ngẫu nhiên trong thuật toán, tuy nhiên không làm thay đổi xu hướng tối ưu tổng thể.

Kết quả này phù hợp với các nghiên cứu trước đây, trong đó Deb và cộng sự [1] đã chứng minh rằng NSGA-II có khả năng duy trì sự đa dạng nghiệm và hội tụ hiệu quả nhờ cơ chế *non-dominated sorting* kết hợp với *crowding distance*. Tương tự, Coello Coello [2] cũng chỉ ra rằng các thuật toán tiến hóa đa mục tiêu thường cho kết quả dao động nhẹ giữa các lần chạy, nhưng vẫn đảm bảo tính ổn định của Pareto front nếu cấu hình thuật toán phù hợp. Ngoài ra, Konak và cộng sự [3] nhấn mạnh rằng việc thực hiện nhiều lần chạy và so sánh các Pareto front là cần thiết để đánh giá độ tin cậy của kết quả tối ưu trong các bài toán đa mục tiêu.

Dựa trên kết quả thu được, lần chạy thứ 2 (seed = 2) được lựa chọn làm tập nghiệm đại diện cho các phân tích tiếp theo, do thể hiện sự phân bố nghiệm mượt và bao phủ tốt toàn bộ không gian mục tiêu. Nhìn chung, các kết quả đạt được khẳng định rằng thuật toán NSGA-II có khả năng tìm kiếm hiệu quả và ổn định các nghiệm Pareto tối ưu cho bài toán tối ưu đa mục tiêu đang xét, phù hợp với các công bố trong lĩnh vực tối ưu tiến hóa.

[1] Deb K, Pratap A, Agarwal S, Meyarivan T.
*A fast and elitist multiobjective genetic algorithm: NSGA-II.*
IEEE Transactions on Evolutionary Computation. 2002;6(2):182–197.
<https://doi.org/10.1109/4235.996017>

[2] Coello Coello CA.
*Evolutionary multi-objective optimization: a historical view of the field.*
IEEE Computational Intelligence Magazine. 2006;1(1):28–36.
https://doi.org/10.1109/MCI.2006.1597059

[3] Konak A, Coit DW, Smith AE.
*Multi-objective optimization using genetic algorithms: A tutorial.*
Reliability Engineering & System Safety. 2006;91(9):992–1007.
https://doi.org/10.1016/j.ress.2005.11.018

Fig. X. Comparison of Pareto fronts obtained from five independent NSGA-II runs.

Done.

Saved: NSGA2_Sigmas_results.mat

Exported: Pareto_Sigmas.xlsx

Saved multi-run results: NSGA2_multi_runs.mat

Best run = 2 (seed = 2)

Saved best run: NSGA2_best_run.mat

Từ tập nghiệm Pareto thu được, điểm knee được xác định dựa trên tiêu chí khoảng cách vuông góc cực đại đến đường nối hai nghiệm biên, nhằm lựa chọn nghiệm có mức cân bằng tối ưu giữa hai mục tiêu. Kết quả cho thấy điểm knee đạt được tại tổ hợp thông số $A={499.998}^{\circ}C$, $B=50.000$kN, $C=18.867$min, $D={16.496}^{\circ}C/min$, và $E=40.005\%$, tương ứng với giá trị $\sigma_{t(\mathrm{Al}-\mathrm{Al})}=208.30$MPa và $\sigma_{t(\mathrm{Al}-\mathrm{Fe}-\mathrm{Al})}=301.58$MPa. Vị trí của điểm này nằm tại vùng cong nhất của Pareto front, nơi sự đánh đổi giữa hai mục tiêu trở nên rõ rệt nhất.

Để đánh giá tính ổn định của nghiệm knee, năm nghiệm Pareto lân cận đã được lựa chọn và trình bày trong Bảng X. Có thể nhận thấy rằng các nghiệm này có giá trị thông số rất gần nhau, đặc biệt là nhiệt độ ép (A ≈ 500°C), lực ép (B = 50 kN) và công suất gia nhiệt (E ≈ 40%), cho thấy đây là vùng tham số tối ưu chi phối mạnh đến cơ tính của vật liệu. Đồng thời, các giá trị $\sigma_{t(\mathrm{Al}-\mathrm{Al})}$và $\sigma_{t(\mathrm{Al}-\mathrm{Fe}-\mathrm{Al})}$trong nhóm nghiệm này chỉ dao động trong khoảng hẹp, chứng tỏ độ nhạy của bài toán quanh điểm tối ưu là thấp và nghiệm knee có tính đại diện cao.

Ngoài ra, có thể quan sát rằng khi dịch chuyển khỏi điểm knee theo hai hướng của Pareto front, sự cải thiện của một mục tiêu sẽ kéo theo sự suy giảm rõ rệt của mục tiêu còn lại. Điều này xác nhận rằng điểm knee thực sự là nghiệm trade-off tối ưu, nơi đạt được sự cân bằng tốt nhất giữa hai tiêu chí. Kết quả này phù hợp với các nghiên cứu trước đây, trong đó Das [1] và Branke et al. [2] đã chỉ ra rằng điểm knee thường đại diện cho nghiệm tối ưu thực tiễn nhất trong các bài toán đa mục tiêu, do cung cấp lợi ích biên lớn nhất so với sự đánh đổi phải chấp nhận.

Hơn nữa, Deb và cộng sự [3] cũng nhấn mạnh rằng việc kết hợp NSGA-II với tiêu chí lựa chọn knee point là một phương pháp hiệu quả để xác định nghiệm tối ưu đại diện từ tập Pareto. Như vậy, việc lựa chọn điểm knee cùng với các nghiệm lân cận trong nghiên cứu này không chỉ đảm bảo tính tối ưu mà còn phù hợp với các phương pháp được công nhận trong lĩnh vực tối ưu đa mục tiêu.

**[1]**Das I.
*On characterizing the “knee” of the Pareto curve based on normal-boundary intersection.*
Structural Optimization. 1999;18:107–115.
https://doi.org/10.1007/BF01195985

**[2]**Branke J, Deb K, Dierolf H, Osswald M.
*Finding knees in multi-objective optimization.*
Parallel Problem Solving from Nature (PPSN). 2004.
https://doi.org/10.1007/978-3-540-30217-9_84

**[3]**Deb K, Pratap A, Agarwal S, Meyarivan T.
*A fast and elitist multiobjective genetic algorithm: NSGA-II.*
IEEE Transactions on Evolutionary Computation. 2002;6(2):182–197.
<https://doi.org/10.1109/4235.996017>

Fig. X. Pareto front and knee point identified using the maximum perpendicular distance method.

=== Knee point ===

A=499.998, B=50.000, C=18.867, D=16.496, E=40.005

Sigma_AlAl=208.296500, Sigma_AlFeAl=301.576381

Bảng kết quả 5 điểm gần điểm tối ưu

| No | A_C | B_kN | C_min | D_Cmin | E_pct | Sigma_AlAl | Sigma_AlFeAl |
| --- | --- | --- | --- | --- | --- | --- | --- |
| 1 | 500 | 50 | 24.9 | 16.4 | 40 | 208.6 | 300.7 |
| 2 | 500 | 50 | 13.1 | 17 | 40 | 207.4 | 302.3 |
| 3 | 500 | 50 | 18.9 | 16.5 | 40 | 208.3 | 301.6 |
| 4 | 500 | 50 | 18.1 | 16.6 | 40 | 208.2 | 301.7 |
| 5 | 500 | 50 | 20 | 16.6 | 40 | 208.4 | 301.5 |
