# Supplementary material for: Optimization of process parameters for enhancing the tensile strength of diffusion-bonded Al–Al and Al–Fe–Al laminated structures
Source: PLoS One. 2026 Aug 11;21(8):e0354878. doi: 10.1371/journal.pone.0354878 (PMC13460570; doi:10.1371/journal.pone.0354878)
Supplement: Peer review history

# Peer review: Decision Letter 0

**DOI:** 10.1371/journal.pone.0354878.r001

**Authors:** Din Bandhu

3 May 2026

Dear Dr. Son Minh,

Please submit your revised manuscript by Jun 17 2026 11:59PM. If you will need more time than this to complete your revisions, please reply to this message or contact the journal office at plosone@plos.org. When you're ready to submit your revision, log on to https://www.editorialmanager.com/pone/ and select the 'Submissions Needing Revision' folder to locate your manuscript file.

- A letter that responds to each point raised by the academic editor and reviewer(s). You should upload this letter as a separate file labeled 'Response to Reviewers'.
- A marked-up copy of your manuscript that highlights changes made to the original version. You should upload this as a separate file labeled 'Revised Manuscript with Track Changes'.
- An unmarked version of your revised paper without tracked changes. You should upload this as a separate file labeled 'Manuscript'.

If you would like to make changes to your financial disclosure, please include your updated statement in your cover letter. Guidelines for resubmitting your figure files are available below the reviewer comments at the end of this letter.

If applicable, we recommend that you deposit your laboratory protocols in protocols.io to enhance the reproducibility of your results. Protocols.io assigns your protocol its own identifier (DOI) so that it can be cited independently in the future. For instructions see: https://journals.plos.org/plosone/s/submission-guidelines#loc-laboratory-protocols. Additionally, PLOS ONE offers an option for publishing peer-reviewed Lab Protocol articles, which describe protocols hosted on protocols.io. Read more information on sharing protocols at https://plos.org/protocols?utm_medium=editorial-email&utm_source=authorletters&utm_campaign=protocols.

As the corresponding author, your ORCID iD is verified in the submission system and will appear in the published article. PLOS supports the use of ORCID, and we encourage all coauthors to register for an ORCID iD and use it as well. Please encourage your coauthors to verify their ORCID iD within the submission system before final acceptance, as unverified ORCID iDs will not appear in the published article. *Only*  the individual author can complete the verification step; PLOS staff *cannot*  verify ORCID iDs on behalf of authors.

We look forward to receiving your revised manuscript.

Kind regards,

Din Bandhu, Ph.D.

Academic Editor

PLOS One

Journal Requirements:

When submitting your revision, we need you to address these additional requirements.

1. Please ensure that your manuscript meets PLOS ONE's style requirements, including those for file naming. The PLOS ONE style templates can be found at

https://journals.plos.org/plosone/s/file?id=wjVg/PLOSOne_formatting_sample_main_body.pdf and

https://journals.plos.org/plosone/s/file?id=ba62/PLOSOne_formatting_sample_title_authors_affiliations.pdf

2. We note that Figure 3 in your submission contain copyrighted images. All PLOS content is published under the Creative Commons Attribution License (CC BY 4.0), which means that the manuscript, images, and Supporting Information files will be freely available online, and any third party is permitted to access, download, copy, distribute, and use these materials in any way, even commercially, with proper attribution. For more information, see our copyright guidelines: http://journals.plos.org/plosone/s/licenses-and-copyright.

We require you to either (1) present written permission from the copyright holder to publish these figures specifically under the CC BY 4.0 license, or (2) remove the figures from your submission:

1. You may seek permission from the original copyright holder of Figure 3 to publish the content specifically under the CC BY 4.0 license.

We recommend that you contact the original copyright holder with the Content Permission Form (http://journals.plos.org/plosone/s/file?id=7c09/content-permission-form.pdf) and the following text:

“I request permission for the open-access journal PLOS ONE to publish XXX under the Creative Commons Attribution License (CCAL) CC BY 4.0 (http://creativecommons.org/licenses/by/4.0/). Please be aware that this license allows unrestricted use and distribution, even commercially, by third parties. Please reply and provide explicit written permission to publish XXX under a CC BY license and complete the attached form.”

Please upload the completed Content Permission Form or other proof of granted permissions as an "Other" file with your submission.

In the figure caption of the copyrighted figure, please include the following text: “Reprinted from [ref] under a CC BY license, with permission from [name of publisher], original copyright [original copyright year].”

2. If you are unable to obtain permission from the original copyright holder to publish these figures under the CC BY 4.0 license or if the copyright holder’s requirements are incompatible with the CC BY 4.0 license, please either i) remove the figure or ii) supply a replacement figure that complies with the CC BY 4.0 license. Please check copyright information on all replacement figures and update the figure caption with source information. If applicable, please specify in the figure caption text when a figure is similar but not identical to the original image and is therefore for illustrative purposes only.

3. Please include captions for your Supporting Information files at the end of your manuscript, and update any in-text citations to match accordingly. Please see our Supporting Information guidelines for more information: http://journals.plos.org/plosone/s/supporting-information.

4. If the reviewer comments include a recommendation to cite specific previously published works, please review and evaluate these publications to determine whether they are relevant and should be cited. There is no requirement to cite these works unless the editor has indicated otherwise.

[Note: HTML markup is below. Please do not edit.]

Reviewers' comments:

Reviewer's Responses to Questions

**Comments to the Author**

1. Is the manuscript technically sound, and do the data support the conclusions?

Reviewer #1: Yes

Reviewer #2: Yes

**********

2. Has the statistical analysis been performed appropriately and rigorously?

Reviewer #1: No

Reviewer #2: Yes

**********

3. Have the authors made all data underlying the findings in their manuscript fully available?

The PLOS Data policy

Reviewer #1: Yes

Reviewer #2: Yes

**********

4. Is the manuscript presented in an intelligible fashion and written in standard English?

Reviewer #1: Yes

Reviewer #2: Yes

**********

Reviewer #1: The manuscript investigates optimization of diffusion bonding parameters for Al–Al and Al–Fe–Al laminated structures using BBD–RSM and NSGA-II. The topic is relevant and technically sound, combining experimental work with statistical and optimization tools. However, while the methodology is structured, the manuscript still lacks depth in scientific interpretation, novelty positioning, and validation rigor, which are critical for publication in a journal like PLOS ONE. Overall, the manuscript would benefit significantly from additional experimental characterization, particularly in terms of phase identification, interfacial analysis, and mechanical testing. These analyses are essential to validate the proposed bonding mechanisms and strengthen the scientific rigor of the study.

1. The manuscript claims limited studies on Al–Al and Al–Fe–Al systems, but this is not convincingly demonstrated. The novelty appears incremental:

BBD + RSM + NSGA-II is already widely used in manufacturing optimization.

2. The authors must clearly state What is new beyond existing diffusion bonding optimization studies? Is it Dual-material optimization? Specific parameter combination? Or Microstructural insight?

3. Include and discuss Recent optimization-based works (2023–2025). Comparative methodologies (ANN, GA, hybrid models)

4. Formula for number of experiments is given, but Why 46 runs specifically? How many center points? Clarify Replication strategy and Experimental error estimation.

5. The literature review is insufficient and lacks depth. More recent (last 3-5 years) references should be incorporated. Cite these papers:

https://journals.plos.org/plosone/article?id=10.1371/journal.pone.0341127

https://www.sciencedirect.com/science/article/pii/S2238785425032867

https://www.taylorfrancis.com/chapters/edit/10.1201/9781003503828-3/recent-advancement-machine-learning-machining-joining-forming-processes-tanmay-tiwari-aswani-kumar-singh-chandra-sekhar-rakurty-rashi-tyagi-gopal-nadkarni

https://www.nature.com/articles/s41598-026-41113-1

6. Results and Discussion show Superficial Interpretation as Trends are described but not deeply explained. Example: “temperature increases strength” expected, not insightful. Add: Diffusion kinetics explanation, Role of oxide breakdown, Intermetallic formation behavior (especially Fe–Al)

7. No reporting of R², Adjusted R², Predicted R² , Residual plots and Model adequacy plots. Model validation is incomplete.

8. Only qualitative discussion on SEM analysis. No Magnification scale details, EDS analysis, Intermetallic identification. Fe₂Al₅, FeAl₃ phase discussion (critical for Al–Fe systems) must be included. In Al–Fe–Al systems, the formation of intermetallic compounds (e.g., Fe₂Al₅, FeAl₃) is well known and critically affects mechanical performance. However, no phase identification techniques such as X-ray diffraction (XRD) or EDS/EDX analysis have been used. The authors should perform phase analysis to confirm the presence and distribution of intermetallic layers.

9. Higher-resolution and more detailed characterization techniques (Hardness, Residual stress) are required to substantiate the conclusions.

10. The thickness of the diffusion layer/intermetallic region is not quantified.

Reviewer #2: After careful reading of the article, I strongly recommend it for major revision due to the following concerns:

1. Abstract is not well drafted. Write a structured abstract mentioning the statistical data, but within the journal’s template. It is advised to follow IMRAD template for Headings in the article.

2. The abstract claims “limited research on Al-Fe-Al laminated structures” but fails to quantify how many prior studies exist. Please specify the gap with concrete citations.

3. NSGA-II optimization is mentioned, yet the abstract does not state whether the predicted optima were experimentally validated. This must be clarified.

4. The introduction cites several review papers but does not highlight any specific contradictory findings from previous diffusion bonding studies. Add a clear statement of unresolved discrepancies.

5. Cite more contemporary studies to strengthen the research gap. In this regards, the authors are encouraged to cite the following recent and highly relevant articles to strengthen the literature background. Incorporation the following works will improve the article’s readability:

• https://doi.org/10.1016/j.rineng.2025.107054

• https://doi.org/10.1016/j.measurement.2025.120114

• https://doi.org/10.1371/journal.pone.0341127

• https://doi.org/10.1007/s12008-023-01473-8

• https://doi.org/10.1016/j.jmrt.2024.06.079

• https://doi.org/10.1504/IJMATEI.2024.144232

6. Also, it will be great if the authors add a summary of literature just after completion of prior state of the art.

7. The hole-pattern structure on the steel layer is described, but its geometry (diameter, depth, spacing) and how it affects mechanical interlocking are not quantified. Provide their details too.

8. The tensile testing section follows ASTM E8/E8M, yet the reported specimen thickness (6 mm) and gauge length (57 mm) do not match standard subsize proportions. Verify and correct them.

9. Five processing parameters are studied, but the range for cooling rate (13–17°C/min) is extremely narrow. Justify why such a small window was chosen.

10. Figure 5 plots use polynomial fitting without reporting R² or RMSE values. Add these goodness-of-fit metrics for each subplot.

11. The discussion attributes weaker Al-Fe bonding to Young’s modulus mismatch, but no quantitative calculation of interfacial shear stress is provided. Include a simple estimate to support the claim.

12. The SEM images are presented without scale bars or magnification labels. Add these for proper interpretation of interfacial features.

13. First of all, it must be “Conclusions”. In the current form, the presented conclusions section doesn’t provide any concrete outcome to the readers. It is suggested to include the limitations as well as the future work in the article.

Overall, the article is good but lacks clarity in its presentation. However, it can be improvised if worked upon. Therefore, I recommend MAJOR REVISION.

**********

what does this mean?). If published, this will include your full peer review and any attached files.

If you choose “no”, your identity will remain anonymous but your review may still be made public.

**Do you want your identity to be public for this peer review?**  For information about this choice, including consent withdrawal, please see our Privacy Policy

Reviewer #1: No

Reviewer #2: No

**********

[NOTE: If reviewer comments were submitted as an attachment file, they will be attached to this email and accessible via the submission site. Please log into your account, locate the manuscript record, and check for the action link "View Attachments". If this link does not appear, there are no attachment files.]

To ensure your figures meet our technical requirements, please review our figure guidelines: https://journals.plos.org/plosone/s/figures

You may also use PLOS’s free figure tool, NAAS, to help you prepare publication quality figures: https://journals.plos.org/plosone/s/figures#loc-tools-for-figure-preparation.

NAAS will assess whether your figures meet our technical requirements by comparing each figure against our figure specifications.

# Author comment: Author response to Decision Letter 1

**DOI:** 10.1371/journal.pone.0354878.r002

29 Jun 2026

Response to Reviewer #1

1. The manuscript claims limited studies on Al–Al and Al–Fe–Al systems, but this is not convincingly demonstrated. The novelty appears incremental: BBD + RSM + NSGA-II is already widely used in manufacturing optimization.

Respond:

Thank you for this important comment. We agree that BBD, RSM, and NSGA-II are established methods and should not be presented as the sole novelty of the work. In the revised manuscript, we have clarified that the main contribution does not lie in the use of these optimization methods alone, but in applying an integrated experimental–statistical–optimization framework to a dual-material bonding problem involving both Al–Al and Al–Fe–Al laminated structures under the same hot-press diffusion bonding conditions. The Abstract, Introduction, NSGA-II optimization section, and Conclusions have been revised to emphasize that the novelty is the simultaneous evaluation and optimization of tensile performance for similar Al–Al bonding and dissimilar Al–Fe–Al laminated bonding, rather than the simple application of BBD–RSM–NSGA-II.

2. The authors must clearly state What is new beyond existing diffusion bonding optimization studies? Is it Dual-material optimization? Specific parameter combination? Or Microstructural insight?

Respond:

Thank you for this helpful suggestion. The novelty has been clarified in the revised manuscript. Specifically, the study is now positioned as a process-oriented investigation of dual laminated material systems, where Al–Al and Al–Fe–Al stacked structures are compared and optimized within the same hot-press diffusion bonding framework. The new contribution is mainly the simultaneous optimization of the tensile strength responses of both bonding systems using a Pareto-based approach, together with practical guidance for selecting suitable hot-press bonding parameters. A clear novelty paragraph has been added at the end of the Introduction to avoid ambiguity regarding the contribution of the work.

3. Include and discuss Recent optimization-based works (2023–2025). Comparative methodologies (ANN, GA, hybrid models)

Respond:

Thank you for the suggestion. We have expanded the Introduction to include recent optimization-based studies in manufacturing and joining processes. The revised literature review now discusses not only RSM-based optimization, but also ANN, GA, PSO, GRA, TOPSIS, and hybrid optimization frameworks. The added studies help place the present work in the context of recent developments in manufacturing optimization and clarify that the novelty of this study is related to the specific dual-material diffusion bonding problem rather than the optimization algorithm alone. Recent optimization-based literature has been integrated into the Introduction, including works such as “Optimization of friction stir welding parameters for dissimilar aluminium alloys using RSM-GRA and RSM-TOPSIS: Towards sustainable manufacturing in industry 4.0,” “Multiobjective optimization of RMD welding parameters for ASTM A387 steel using Taguchi method and artificial neural networks,” “Predicting the thermal performance of screen mesh wick heat pipe with alumina nanofluids using response surface methodology,” “Optimizing wire electrical discharge machining performance of Inconel 625 with genetic algorithms & particle swarm optimization,” and “Data-driven machine learning modelling in wire EDM of TiNiCo shape memory alloy.”

The correction has been made in Page 4.

4. Formula for number of experiments is given, but Why 46 runs specifically? How many center points? Clarify Replication strategy and Experimental error estimation.

Respond:

Thank you for pointing this out. The section “Box–Behnken Design (BBD) and Analysis of Variance (ANOVA)” has been revised to clarify why 46 experimental runs were used. For a five-factor Box–Behnken Design, 40 non-center design points are required according to the BBD formulation. In addition, six replicated center points were included to estimate pure experimental error and evaluate process repeatability. Therefore, the total number of experimental runs was 46. The revised manuscript now also explains that these center-point replications support the lack-of-fit analysis and provide a basis for evaluating the stability of the experimental process.

The correction has been made in Page 10.

5. The literature review is insufficient and lacks depth. More recent (last 3-5 years) references should be incorporated. Cite these papers:

Respond:

Thank you for this valuable suggestion. The Introduction has been substantially revised to strengthen the literature background and research gap. We have incorporated recent studies on aluminum alloys, aluminum-based composites, Al–Fe bonding, Fe–Al intermetallic behavior, solid-state joining, and optimization-based manufacturing methods. The reviewer-suggested references have been added and discussed in the revised Introduction, including “Synergistic enhancement of Al-Si7Mg alloy: Strengthening mechanical properties through combined electromagnetic agitation and AL-10% Ti refinement,” “Additive Manufacturing of Al-Based Metal Matrix Composites: A Review with Bibliometric Perspective on Biomedical Applications,” and “Data-driven machine learning modelling in wire EDM of TiNiCo shape memory alloy.” These additions improve the depth of the literature review and help clarify the modern relevance and research gap of the present study.

The correction has been made in Page 2, 3.

6. Results and Discussion show Superficial Interpretation as Trends are described but not deeply explained. Example: “temperature increases strength” expected, not insightful. Add: Diffusion kinetics explanation, Role of oxide breakdown, Intermetallic formation behavior (especially Fe–Al)

Respond:

Thank you for this important comment. The Results and Discussion section has been revised to provide a deeper physical interpretation of the observed trends. The effects of pressing temperature and pressing force are now explained in terms of enhanced atomic mobility, improved real contact area, plastic deformation of surface asperities, reduced interfacial voids, and possible disruption of the aluminum oxide layer. For the Al–Fe–Al system, the discussion now includes the possible role of Fe–Al intermetallic compounds based on previous literature. However, because EDS/XRD analysis was not performed in the present study, the manuscript has been carefully revised to avoid unsupported claims regarding direct phase identification. The sections “Box–Behnken Design (BBD) and Analysis of Variance (ANOVA),” “NSGA-II multi-objective optimization,” and “SEM image analysis” have all been revised to improve the physical interpretation of the statistical and SEM results. In addition, a boxplot was added to better visualize the experimental dataset before detailed statistical modeling.

The correction has been made in Page 16-25.

7. No reporting of R², Adjusted R², Predicted R² , Residual plots and Model adequacy plots. Model validation is incomplete.

Respond:

Thank you for this important suggestion. The ANOVA and modeling section has been revised to include additional model adequacy information. The revised manuscript now reports and discusses R² and adjusted R² values for both Al–Al and Al–Fe–Al tensile strength models. For the Al–Al model, R² and adjusted R² are 71.66% and 48.98%, respectively. For the Al–Fe–Al model, R² and adjusted R² are 75.00% and 55.00%, respectively. These values are interpreted cautiously, and the manuscript now states that the models are statistically useful for process trend analysis and optimization within the studied range, but their predictive capability is moderate. In addition, predicted-versus-experimental parity plots were added, together with R² and RMSE values, to further evaluate model prediction performance.

The correction has been made in Page 20 – 22

8. Only qualitative discussion on SEM analysis. No Magnification scale details, EDS analysis, Intermetallic identification. Fe₂Al₅, FeAl₃ phase discussion (critical for Al–Fe systems) must be included. In Al–Fe–Al systems, the formation of intermetallic compounds (e.g., Fe₂Al₅, FeAl₃) is well known and critically affects mechanical performance. However, no phase identification techniques such as X-ray diffraction (XRD) or EDS/EDX analysis have been used. The authors should perform phase analysis to confirm the presence and distribution of intermetallic layers.

Respond:

Thank you for this valuable comment. We agree that EDS, XRD, and diffusion-layer thickness measurements would provide deeper insight into the interfacial reaction and possible Fe–Al intermetallic formation. However, these characterization facilities were not available for the present study. Therefore, the manuscript has been revised to avoid unsupported claims regarding phase identification, intermetallic distribution, or diffusion-layer thickness. The SEM analysis is now presented only as qualitative evidence of interfacial morphology and fracture behavior. The revised text clearly states that EDS/XRD/TEM analysis and diffusion-layer thickness measurement were not performed. Fe–Al intermetallic compounds such as Fe₂Al₅ and FeAl₃ are now discussed only as possible literature-based phenomena, not as direct experimental evidence from the present work. This limitation has also been explicitly stated in the Conclusions, and future work has been suggested to include elemental mapping, phase identification, and quantitative interfacial layer measurement.

The correction has been made in Page 27-28.

9. Higher-resolution and more detailed characterization techniques (Hardness, Residual stress) are required to substantiate the conclusions.

Respond:

Thank you for the suggestion. We agree that additional characterization, such as hardness distribution and residual stress analysis, would further strengthen the interpretation of the bonding behavior. However, these measurements were outside the available experimental scope of the present study. To address this limitation, the manuscript has been revised to state more clearly that the present work mainly evaluates bonding performance based on tensile strength and qualitative SEM observation. The Conclusions section has also been expanded to acknowledge the absence of hardness and residual stress measurements and to suggest these analyses as important directions for future work, together with shear strength, fatigue testing, and more detailed interfacial characterization.

The correction has been made in Page 26-28.

10. The thickness of the diffusion layer/intermetallic region is not quantified.

Respond:

Thank you for this comment. We agree that quantifying the diffusion layer or intermetallic region would be valuable for understanding the detailed bonding mechanism, particularly in the Al–Fe–Al laminated structure. However, due to the absence of EDS/XRD/TEM analysis and suitable interfacial measurement facilities, the diffusion-layer or intermetallic-region thickness could not be quantified in the present study. The revised SEM section now clearly states this limitation and avoids making unsupported claims about the thickness or distribution of intermetallic compounds. The Conclusions section has also been revised to identify quantitative interfacial layer measurement as an important future research direction.

The correction has been made in Page 26-28.

Response to Reviewer #2

1. Abstract is not well drafted. Write a structured abstract mentioning the statistical data, but within the journal’s template. It is advised to follow IMRAD template for Headings in the article.

Respond:

Thank you for this helpful comment. The Abstract has been revised to improve its clarity, structure, and focus. The revised Abstract now follows a clearer background–gap–method–result–contribution flow and better reflects the main content of the manuscript. It emphasizes the hot-press diffusion bonding process, dual-material optimization of Al–Al and Al–Fe–Al structures, BBD–RSM modeling, NSGA-II optimization, and tensile performance. In addition, the manuscript has been reformatted according to the journal template, including the use of appropriate heading levels. The revised Abstract also avoids overclaiming detailed diffusion-mechanism analysis because EDS/XRD and diffusion-layer thickness measurements were not performed in the present study.

2. The abstract claims “limited research on Al-Fe-Al laminated structures” but fails to quantify how many prior studies exist. Please specify the gap with concrete citations.

Respond:

Thank you for this comment. The Abstract and Introduction have been revised to clarify the research gap more carefully and to support the statement with more specific literature discussion. Instead of making a broad unsupported claim, the revised manuscript now explains that most previous studies have focused on direct Al–Fe interfaces, interlayer-assisted Al–Fe bonding, or friction-based Al–Fe joining, whereas fewer studies have addressed Al–Fe–Al laminated structures in which steel serves as the core layer and aluminum acts as the outer layers (No research has investigated this issue). Recent studies were added to strengthen this point and to show that the present work specifically investigates Al–Fe–Al laminated structures and the simultaneous optimization of Al–Al and Al–Fe–Al systems under the same hot-press diffusion bonding framework.

The correction has been made in Page 1 and Introduction part.

3. NSGA-II optimization is mentioned, yet the abstract does not state whether the predicted optima were experimentally validated. This must be clarified.

Respond:

Thank you for this important comment. We have clarified this issue in the revised manuscript. The NSGA-II predicted optimum was experimentally verified, and the validation results have now been added on page 25 of the revised manuscript, immediately after the Pareto front and knee-point optimization results. Specifically, confirmation experiments were conducted using the optimized processing parameters obtained from the knee-point solution: pressing temperature of 500 °C, pressing force of 50 kN, holding time of 18.87 min, cooling rate of 16.50 °C/min, and heating power of 40%.

The predicted tensile strengths obtained from the RSM–NSGA-II model were 208.30 MPa for the Al–Al bonded structure and 301.58 MPa for the Al–Fe–Al laminated structure. The corresponding experimental values were 208.50 MPa and 299.70 MPa, respectively. The deviations between predicted and experimental values were only 0.10% for Al–Al and 0.62% for Al–Fe–Al. These low deviations confirm that the predicted optimum was experimentally validated and that the RSM–NSGA-II model can reasonably predict the tensile strength responses within the investigated design space.

4. The introduction cites several review papers but does not highlight any specific contradictory findings from previous diffusion bonding studies. Add a clear statement of unresolved discrepancies.

Respond:

Thank you for this valuable comment. The Introduction has been revised to include a more critical discussion of previous findings, particularly regarding Fe–Al intermetallic compounds in Al–Fe bonding systems. The revised manuscript now explains that some studies reported that thin or discontinuous intermetallic layers may not severely reduce bonding strength, whereas thick, continuous, or cracked intermetallic layers can significantly deteriorate interfacial performance. This unresolved discrepancy was added to highlight that the effect of Fe–Al intermetallic compounds depends not only on their presence, but also on their thickness, morphology, continuity, and crack formation. This discussion helps strengthen the motivation for investigating the process–property relationship in Al–Al and Al–Fe–Al laminated structures.

The correction has been made in Page 3.

5. Cite more contemporary studies to strengthen the research gap. In this regards, the authors are encouraged to cite the following recent and highly relevant articles to strengthen the literature background. Incorporation the following works will improve the article’s readability:

Respond:

Thank you for this valuable suggestion. We agree that incorporating more recent and relevant studies can strengthen the literature backgr

Attachment Submitted filename: Reply _ Reviewer 2.docx

# Peer review: Decision Letter 1

**DOI:** 10.1371/journal.pone.0354878.r003

**Authors:** Din Bandhu

14 Jul 2026

Optimization of Process Parameters for Enhancing the Tensile Strength of Diffusion-Bonded Al–Al and Al–Fe–Al Laminated Structures

PONE-D-26-15896R1

Dear Dr. Son Minh,

We’re pleased to inform you that your manuscript has been judged scientifically suitable for publication and will be formally accepted for publication once it meets all outstanding technical requirements.

Within one week, you’ll receive an e-mail detailing the required amendments. When these have been addressed, you’ll receive a formal acceptance letter and your manuscript will be scheduled for publication.

An invoice will be generated when your article is formally accepted. Please note, if your institution has a publishing partnership with PLOS and your article meets the relevant criteria, all or part of your publication costs will be covered. Please make sure your user information is up-to-date by logging into Editorial Manager at Editorial Manager® and clicking the ‘Update My Information' link at the top of the page. For questions related to billing, please contact billing support.

If your institution or institutions have a press office, please notify them about your upcoming paper to help maximize its impact. If they’ll be preparing press materials, please inform our press team as soon as possible -- no later than 48 hours after receiving the formal acceptance. Your manuscript will remain under strict press embargo until 2 pm Eastern Time on the date of publication. For more information, please contact onepress@plos.org.

Kind regards,

Din Bandhu, Ph.D.

Academic Editor

PLOS One

Additional Editor Comments (optional):

Reviewers' comments:

Reviewer's Responses to Questions

**Comments to the Author**

Reviewer #1: All comments have been addressed

Reviewer #2: All comments have been addressed

**********

2. Is the manuscript technically sound, and do the data support the conclusions?

Reviewer #1: Yes

Reviewer #2: Yes

**********

3. Has the statistical analysis been performed appropriately and rigorously?

Reviewer #1: Yes

Reviewer #2: Yes

**********

4. Have the authors made all data underlying the findings in their manuscript fully available?

The PLOS Data policy

Reviewer #1: Yes

Reviewer #2: Yes

**********

5. Is the manuscript presented in an intelligible fashion and written in standard English?

Reviewer #1: Yes

Reviewer #2: Yes

**********

Reviewer #1: I recommend acceptance of the manuscript for publication. Prior to publication, the authors may consider carefully proofreading the manuscript to correct a few minor grammatical and typographical errors, improve consistency in terminology and units, and update the literature review with a few recent relevant references where appropriate to further strengthen the discussion.

Reviewer #2: All comments have been addressed by the authors. Hence, I recommend for the acceptance of this article.

**********

what does this mean?). If published, this will include your full peer review and any attached files.

If you choose “no”, your identity will remain anonymous but your review may still be made public.

**Do you want your identity to be public for this peer review?**  For information about this choice, including consent withdrawal, please see our Privacy Policy

Reviewer #1: No

Reviewer #2: No

**********

# Editor report: Acceptance letter

**DOI:** 10.1371/journal.pone.0354878.r004

**Authors:** Din Bandhu

PONE-D-26-15896R1

PLOS One

Dear Dr. Son Minh,

I'm pleased to inform you that your manuscript has been deemed suitable for publication in PLOS One. Congratulations! Your manuscript is now being handed over to our production team.

At this stage, our production department will prepare your paper for publication. This includes ensuring the following:

* All references, tables, and figures are properly cited

* All relevant supporting information is included in the manuscript submission,

* There are no issues that prevent the paper from being properly typeset

You will receive further instructions from the production team, including instructions on how to review your proof when it is ready. Please keep in mind that we are working through a large volume of accepted articles, so please give us a few days to review your paper and let you know the next and final steps.

Lastly, if your institution or institutions have a press office, please let them know about your upcoming paper now to help maximize its impact. If they'll be preparing press materials, please inform our press team within the next 48 hours. Your manuscript will remain under strict press embargo until 2 pm Eastern Time on the date of publication. For more information, please contact onepress@plos.org.

You will receive an invoice from PLOS for your publication fee after your manuscript has reached the completed accept phase. If you receive an email requesting payment before acceptance or for any other service, this may be a phishing scheme. Learn how to identify phishing emails and protect your accounts at https://explore.plos.org/phishing.

If we can help with anything else, please email us at customercare@plos.org.

Thank you for submitting your work to PLOS ONE and supporting open access.

Kind regards,

PLOS ONE Editorial Office Staff

on behalf of

Dr. Din Bandhu

Academic Editor

PLOS One
